# A Novel Thin-Film Technique to Improve Accuracy of Fluorescence-Based Estimates for Periphytic Biofilms

**DOI:** 10.3390/w13111464

**Published:** 2021-05-23

**Authors:** Leon Katona, Yvonne Vadeboncoeur, Christopher T. Nietch, Katie Hossler

**Affiliations:** 1 Department of Biological Sciences, Wright State University, 3640 Colonel Glenn Hwy, Dayton, OH 43435, USA;; 2 USEPA Office of Research and Development, Center for Environmental Measurement and Modeling, 26 W Martin Luther King Dr, Cincinnati, OH 45268, USA

**Keywords:** periphyton, biofilm, pulse-amplitude modulated fluorometry, photophysiology, photosynthetic efficiency

## Abstract

Recent studies suggest that photophysiological parameters for intact substrates with depth (e.g., periphytic biofilms, microphytobenthos) are overestimated by pulse-amplitude modulated (PAM) fluorometry. This overestimation results from depth-integration effects, following the activation of deeper photosynthesizing layers by an attenuated light signal. To mitigate this error, we propose a novel slide-based thin-film technique in which fluorescence is measured on a vertically representative subsample of the biofilm, spread evenly on a microscope slide. We compared bias and precision for photosynthetic parameters estimated through conventional PAM fluorometry on intact biofilms and through our novel slide-based technique, both theoretically and empirically. Numerical simulations confirmed the consistent overestimation of key parameters for intact biofilms, with relative errors up to 145%, compared to, at most, 52% on thin films. Paired empirical observations likewise demonstrated that estimates based on intact biofilms were consistently higher (up to 248%, *p* < 0.001) than estimates from thin films. Numerical simulation suggested greater precision with the slide-based technique for homogeneous biofilms, but potentially less precision for heterogeneous biofilms with improper subsampling. Our empirical comparison, however, demonstrated some improvement in precision with the slide-based technique (e.g., the coefficient of variation for the maximum electron transport rate was reduced 30%, *p* = 0.009). We recommend the use of the slide-based technique, particularly for biofilms that are thick or have small light attenuation coefficients. Care should be taken, however, to obtain vertically representative subsamples of the biofilm for measurement.

## 1. Introduction

Pulse-amplitude modulated (PAM) fluorometry has emerged as a vital tool to assess physiological efficiencies of photosynthesizing organisms and communities. The technique is both rapid and non-destructive and measures chlorophyll fluorescence in response to increasing levels of irradiance. These measurements yield relative electron transport rate (rETR) profiles over irradiance, or rapid light curves (RLCs), which are very similar in form to photosynthesis–irradiance (P–E) curves. Common photophysiological parameters estimated from the RLCs include the maximum electron transport rate (*ETR*_*m*_), the minimum saturating irradiance (*E*_*k*_), and the photosynthetic efficiency (*α*) (e.g., [1–3]).

Various studies, however, have demonstrated theoretically and experimentally, that estimates of the photophysiological parameters (particularly *ETR*_*m*_ and *E*_*k*_) are subject to overestimation when the photosynthesizing sample has depth or optical density (e.g., epilithic biofilm, microphytobenthos, thick plant tissue), in contrast to diffuse suspensions or isolates obtained on lens tissue [4–7]. For samples with depth, the experimentally measured fluorescence includes surface layers, as well as deeper layers responding to lower irradiance because of the vertical attenuation of light. The inability to correctly account for the influence of the photosynthetic pigments at the different depths due to the attenuation of light (“depth-integration effects”) results in the overestimation of the calculated effective quantum yield (Φ_*PSII*_) and corresponding rETR at higher irradiance. The possibility of depth-integration effects is particularly problematic when assessing the photophysiology of intact biofilms, which typically have thicknesses on the order of millimeters [8,9].

In addition to the misestimation of photophysiological parameters, the effect of depth integration has the potential to confound bioassays or similar impact studies where biofilm thickness might vary with condition. PAM fluorometry has frequently been employed to monitor and assess ecotoxicological impacts [10–12] and nutrient limitation [13]. Bouma-Gregson et al. [14] used PAM fluorometry to evaluate impacts of stream depth, temperature and flow on photosynthetic parameters. If care is not taken to control for differences in biofilm thickness, estimates of photophysiological impact based on condition (e.g., toxin or nutrient exposure, stream flow) will be biased. For example, if toxin exposure also decreases biofilm biomass and thickness, any impacts on photophysiology will likely be overestimated (e.g., absence of toxin will result in thicker biofilms and the overestimation of photophysiology which would result in an apparent greater magnitude of toxin impact).

In contrast, thin layers or optically dilute samples (e.g., thin biofilms, dilute algal suspension, plant tissue with only a few cell layers) can be measured without bias for the photophysiological parameters. When this is not possible, methods to correct for error induced by depth integration include deconvolution of the Φ_*PSII*_–E curves [4]; use of an imaging-PAM which tends to activate only the uppermost layers [6]; and use of fine-tuned techniques that measure fluorescence profiles at variable depths using a fiber-optic-based PAM [15,16] or tissue cross sections and microscopy setups [7].

Herein, we present an alternative technique to improve accuracy in the estimation of photophysiological parameters, particularly for periphytic biofilms. With this technique, a vertically representative subsample of a biofilm (i.e., as much as possible, the different layers in the biofilm are proportionately represented in the subsample) is spread evenly on a microscope slide, thus creating a thin film which is then assessed using a conventional PAM fluorometer. The thin film ensures that the entire subsample will receive the applied irradiance, rather than some portions (i.e., those deeper in the biofilm) receiving attenuated irradiance. Thus, this “slide-based” method should avoid the depth-integration artifacts anticipated if instead the intact biofilm were to be assessed (“intact-biofilm” method). Additionally, the proposed technique is relatively easy to implement with a standard PAM fluorometer and is suitable for measurements in the lab or in the field.

We use the depth-integration model presented in Serôdio [4] to test comparative predictions of bias and precision for both the new slide-based method and the traditional intact-biofilm method. For this theoretical assessment, we model both homogeneous-photic communities (i.e., photophysiological parameters are identical throughout the vertical structure of the biofilm; this is similar to Serôdio [4], but we assess both high-light-adapted and low-light-adapted communities of limited thickness) and heterogeneous-photic communities (i.e., photophysiological parameters vary based on vertical position in the biofilm). Following Serôdio [4], we estimate measured photophysiologies of biofilms with thickness (intact-biofilm method) and compare to “true” photophysiologies (typically, the slide-based method). We use these estimates to calculate bias and precision. We then apply both the slide-based and the intact-biofilm method to natural biofilms that had colonized gravel and artificial tile substrates during a continuous flow-through stream mesocosm experiment for an empirical pair-wise evaluation of bias and precision.

For bias (i.e., closeness to truth), we hypothesized that the slide method would be less biased than the intact-biofilm method. This is expected because of inaccuracies in photophysiological estimates incurred through depth-integration effects. It has been demonstrated theoretically and experimentally that biofilm thickness impacts the PAM fluorescence signal and therefore estimates of Φ_*PSII*_ and rETR through (1) downward vertical attenuation of the measuring and actinic light source; (2) upward vertical attenuation of the fluorescence signal; and (3) vertical heterogeneity in the biofilm (see also Section 2.1.1 [4,5,9,17]). The depth-independent slide method should eliminate much of this inaccuracy, assuming that any vertical heterogeneity is accounted for in the subsampling procedure and preparation of the thin film.

For precision (i.e., replicate variability), we hypothesized that the slide method would be more precise than the intact-biofilm method. For the intact-biofilm, parameter estimates will have errors as a result of depth-integration—the magnitude of which should depend on the biofilm thickness. Spatial variability in thickness across replicates is then expected to result in measurement variability. The slide method, because it is a depth-independent measurement, should eliminate measurement variability associated with varying biofilm thickness. The slide method, however, which also requires the subsampling of the biofilm, might instead introduce measurement variability if there is variability in composition across the replicate subsamples. Other sources of variability are expected to be similar between the two methods (e.g., algal composition, user-/instrument-induced measurement error). We first tested our hypotheses numerically through simulations based on the depth integration model presented in Serôdio [4].

## 2. Materials and Methods

### 2.1. Theoretical Evaluation of Bias and Precision

Following the depth-integration model described in Serôdio [4], we performed a series of simulations to compare the bias and precision of fluorescence-based physiological estimates between the novel proposed slide-based method (i.e., thin film, depth-independent) and the traditional intact-biofilm method (i.e., depth-dependent). The model in Serôdio [4] estimates the distortions in the received fluorescence signals from “thick” substrates (e.g., vertical depth of fractional to several millimeters) due to depth-integration effects. The distortion in the signals typically results in the overestimation of the photophysiological parameters *ETR*_*m*_ and *E*_*k*_. It should be noted that the model in Serôdio [4] primarily describes the expected error, but does not remedy the error (although Serôdio [4] does separately describe a numerical technique to deconvolute fluorescence measurements). We use the model to estimate photophysiological parameters for intact biofilms to compare to “true” values—typically the photophysiological parameters expected from thin film measurements.

#### 2.1.1. The Model

The model presented in Serôdio [4] allows for depth effects implicit in the intact-biofilm method. Note, that although Serôdio [4] was modeling biofilms in sediments (microphytobenthos), the same general model was applicable here for biofilms on surfaces. In the model, fluorescence is estimated at each depth in a biofilm profile based on a depth-independent fluorescence profile *F*(*E*, *z*, *k*_*d*_), which is a function of the surface irradiance *E*, the depth *z* and the attenuation coefficient *k*_*d*_ of the downwelling irradiance. The surface irradiance is attenuated by simple exponential decay as $E\left(z,k_{d}\right)=Ee^{-zk_{d}}$. Modeling the fluorescence that would be measured at the surface of a biofilm (e.g., intact-biofilm method), then simply requires integration over the depth of the biofilm with additional upwelling attenuation of the fluorescence, i.e.,

(1) $$F_{d}\left(E,z,k_{d},k_{u}\right)=\int_{z=0}^{z_{\text{max}}}e^{-zk_{u}}F\left(E,z,k_{d}\right)dz,$$

where the subscript *d* denotes the depth integration of the fluorescence through a biofilm of total thickness *z*_*max*_ (with *z* = 0 denoting the biofilm surface and *z*_*max*_ denoting the maximum depth); *k*_*u*_ is the attenuation coefficient for the upwelling fluorescence; and *F*_*d*_(*E*, *z*, *k*_*d*_, *k*_*u*_) is the measured fluorescence at the surface with depth-integration effects. For further details, refer also to Serôdio [4], particularly Equations (2) and (3). Like Serôdio [4], we made the assumption that vertical attenuation of the downwelling measuring and actinic light would be the same (i.e., same *k*_*d*_; the measuring light is typically of very low intensity, and is sufficient to induce fluorescence but not photosynthesis, whereas the actinic light is of higher and increasing intensity that should induce photosynthesis (e.g., [3])).

Note that the original model also includes a chlorophyll-concentration-dependent and depth-based absorption coefficient *a*_*meas*_(*z*) and a unit conversion factor *G*. In subsequent calculations (e.g., effective quantum yield, Equation (2)), the factor *G* ultimately cancels out. The depth-dependent *a*_*meas*_(*z*), technically does not, but we chose to model as vertically homogeneous (see e.g., Profile C0 in Serôdio [4]), in which case it also ultimately cancels out. Clearly, chlorophyll concentration and absorption can vary with depth [9,18] and will impact the magnitude of depth-integration error [4]. The vertically homogeneous chlorophyll distribution, however, provides a median estimate of depth-integration error, whereas biofilms with chlorophyll concentrated at depth will exaggerate the error and biofilms with chlorophyll concentrated near the surface will mitigate the error (see Serôdio [4], specifically Profiles C2 and C1).

In PAM fluorometry, fluorescence emission is measured over a range of irradiances, with two measures per irradiance: fluorescence following the actinic light application (simply *F*, or the minimum fluorescence yield) and fluorescence following application of a saturating pulse of light ($F_{m^{\prime }}^{\prime }$ or the maximum fluorescence yield). We used the general equation (Equation (1)) to model both: for the actinic light, the functions *F*_*d*_(*E*, *z*, *k*_*d*_, *k*_*u*_) and *F*(*E*, *z*, *k*_*d*_) remain the same; for the saturating light, we denote the functions as *F*_*m*,*d*_(*E*, *z*, *k*_*d*_, *k*_*u*_) and *F*_*m*_(*E*, *z*, *k*_*d*_). The two measures *F* and $F_{m}^{\prime }$ (or *F*_*d*_ and $F_{m,d}^{\prime }$) are combined to estimate the effective quantum yield (Φ_*PSII*_), which is the proportion of incident light that is used for photochemistry via photosystem II (PSII)

(2) $$\Phi_{\text{PSII}}=\frac{\Delta F}{F_{m}^{\prime }}=\frac{{F^{\prime }}_{m}-F}{F_{m}^{\prime }}=1-\frac{F}{F_{m}^{\prime }}.$$

It is the response of this estimate (or the variant *rETR* = *E*Φ_*PSII*_) over the range of irradiance that enables the determination of key photophysiological parameters such as *ETR*_*m*_, *E*_*k*_ and *α*.

The fluorescence yields for the actinic light and the saturating pulse (*F* and $F_{m}^{\prime }$) differ over the range of irradiance (e.g., [4]). This creates a nonlinear relationship of Φ_*PSII*_ to irradiance and additionally makes it non-trivial to correct for the effects of depth-integration. It should be noted, however, that it is primarily the dependence of fluorescence yield on irradiance and the attenuation of irradiance with depth that causes the distortion of Φ_*PSII*_ and *rETR* for thick biofilms (and similar substrates).

In the absence of the downward attenuation of the actinic and saturating pulse of light, Equation (1) becomes

(3) $$F_{d}\left(E,z,k_{u}\right)=\int_{z=0}^{z_{\text{max}}}e^{-zk_{u}}F(E)dz=F(E)\int_{z=0}^{z_{\text{max}}}e^{-zk_{u}}dz.$$

Applying Equation (3) to the actinic light and saturating pulses to obtain *F*_*d*_ and $F_{m,d^{\prime }}^{\prime }$ then substituting into Equation (2), yields

(4) $$\Phi_{\text{PSII}}(E)=1-\frac{F(E)\int_{z=0}^{z_{\text{max}}}e^{-zk_{u}}dz}{F_{m}^{\prime }(E)\int_{z=o}^{z_{\text{max}}}e^{-zk_{u}}dz}=1-\frac{F(E)}{F_{m}^{\prime }(E)}.$$

The effect of upwelling attenuation cancels out and Φ_*PSII*_ (and thus *rETR*) is left undistorted.

#### 2.1.2. Generation of Synthetic Data

Homogeneous biofilms.

We simulated three photosynthetically homogeneous biofilms. Following Serôdio [4], we used measured fluorescence ($F_{m}^{\prime }$ and *F*) over *E* to simulate a thin-film diatom sample (i.e., depth-independent). We used fluorescence patterns in (Figure 2a,b, Serôdio [4]) as a model for a low-light (LL) adapted biofilm (Figure S1, top left), as the $F_{m}^{\prime }$ and *F* response curves matched those observed by Ralph and Gademann [3] for an LL-adapted sea grass (*Zostera marina*; see (Figure 2b, Ralph and Gademann [3])). We additionally modeled two high-light (HL) adapted biofilms. For the HL-adapted biofilms, we based the *F* response curve on that of the HL-adapted sea grass in (Figure 2a, Ralph and Gademann [3]), which was somewhat constant over irradiance. For the HL-adapted $F_{m}^{\prime }$ response curves, we first calculated the LL-adapted *rETR* curve (*rETR* = Φ_*PSII*_ *E*), then adjusted the *rETR* profile to achieve the target parameters of similar *ETR*_*m*_, higher *E*_*k*_ and lower *α* for HL1; and higher *ETR*_*m*_ and *E*_*k*_, and lower *α* for HL2. The simulated response curves can be seen in Figure S1.

Estimates of *ETR*_*m*_, *E*_*k*_, and *α* based on the simulated curves are provided in Table 1. For these estimates, we fit the data to the Jassby and Platt [19] P–E hyperbolic tangent model where *rETR* = *ETR*_*m*_ tanh(*E*/*E*_*k*_). Another common model is the P–E exponential model, where $\text{rETR}=ETR_{m}\left(1-e^{-E/E_{k}}\right)$. This latter model was originally proposed by Webb et al. [20] and is identical to the Platt et al. [21] model when the photoinhibition parameter (*β*) is assumed to be 0. The hyperbolic tangent model provided better fit of the data than the exponential model (not shown).

Heterogeneous biofilms. We simulated two photosynthetically heterogeneous biofilms. To simulate the scenario of a heterogeneous biofilm, we assumed a biofilm composed of an upper HL-adapted layer (either HL1 or HL2; Table 1) and a lower LL-adapted layer, in equal proportions (i.e., *p*_*i*_ = 0.5). We assumed the true *rETR* profile over *E* would be the weighted average of the HL and LL *rETR* profiles (e.g., Figure S2).

More specifically, for a heterogeneous biofilm composed of multiple layers (or species) of differing photophysiologies, we expected the community photophysiological response at a given irradiance to be the weighted average of the photophysiological responses of the component layers, i.e.,

(5) $$\text{rETR}(E)=\overset{N}{\underset{i=1}{\sum }}\left(p_{i}rETR_{i}(E)\right),$$

where *rETR* is the biofilm relative ETR at irradiance *E*; *rETR*_*i*_ is the relative ETR for layer *i* that comprises a proportion *p*_*i*_ (by mass or volume; note also that $\sum_{i}^{N}P_{i}=1$) of the biofilm; and *N* is the total number of layers in the biofilm. The photophysiological parameters (*ETR*_*m*_, *E*_*k*_ and *α*) for the entire biofilm must be solved by fitting the biofilm *rETR* response curve (i.e., weighted-average *rETR*(*E*), Equation (5)) to an appropriate P–E model (e.g., hyperbolic tangent) as the nature of the models do not allow a simple combination of the layer-based photophysiological parameters, e.g., *ETR*_*m*_ ≠ Σ(*p*_*i*_ *ETR*_*m*,*i*_). (Note, however, that in the case of *ETR*_*m*_, simple combination will be a close approximation. If we correctly sum *rETR* response curves across the layers, we get $\text{rETR}(E)=\sum_{i=1}^{N}\left(p_{i}ETR_{m,i}\text{tanh}\left(E/E_{k,i}\right)\right)$; as *E* becomes large $\text{rETR}\approx \sum_{i=1}^{N}\left(p_{i}ETR_{m,i}\right)$, because tanh ∞ → 1.)

To estimate the true photophysiological parameters for the two heterogeneous biofilms, we first averaged the respective *rETR* profiles. The true parameters were then estimated by fitting the averaged *rETR* profiles (Figure S2) to the hyperbolic tangent model (see Table 1). It is noted, however, that the simple averaging of the homogeneous parameters would have resulted in approximately ‘true’ estimates for HL1LL and HL2LL: within 3%, 8% and 11% of *ETR*_*m*_, *E*_*k*_ and *α*, respectively (Table 1).

The actual measured *rETR* over *E* for such a biofilm will differ because it is based on the ratio of two fluorescence yields *F* and $F_{m}^{\prime }$ (i.e., one following application of the actinic light and one following application of the saturating pulse) that will be weighted averages (we will denote as *F*_(*wavg*)_ and $F_{m(\text{wavg})}^{\prime }$ to indicate that these are weighted average fluorescence yields). These two measures are then combined into the effective quantum yield Φ_*PSII*_ for the biofilm (we will denote as Φ_*PSII*(*obs*)_ to indicate that this will be the observed effective quantum yield), and it can be shown that this will not equal the weighted average of the component effective quantum yields, i.e.,

(6) $$F_{\left(\text{wavg}\right)}=\overset{N}{\underset{i=1}{\sum }}\left(p_{i}F_{i}\right)$$

(7) $$F_{m(\text{wavg})}^{\prime }=\overset{N}{\underset{i=1}{\sum }}\left(p_{i}F_{m,i}^{\prime }\right)$$

then,

(8) $$\Phi_{\text{PSII}(obs)}=\frac{F_{m(\text{wavg})}^{\prime }-F_{\left(\text{wavg}\right)}}{F_{m(\text{wavg})}^{\prime }}$$

and,

$$\Phi_{\text{PSII}(obs)}\neq \overset{N}{\underset{i=1}{\sum }}\left(p_{i}\Phi_{\text{PSII},i}\right).$$

Consequently, the actual measured *rETR* over *E* (i.e., *rETR*_(*obs*)_ = *E*Φ_*PSII*(*obs*)_) for a heterogeneous biofilm will differ from the weight-averaged *rETR* (Equation (5)), the latter of which we assume to represent the true photophysiological community response. This discrepancy will factor into predictions of bias for the slide-based method.

#### 2.1.3. Numerical Simulations for Bias and Precision

To compare the two methods based on bias and precision, we explored the impact of biofilm thickness on estimates of *ETR*_*m*_, *E*_*k*_ and *α* for the three photosynthetically homogeneous biofilms and the two photophysiologically heterogeneous biofilms (Figures S1 and S2; Table 1). We first applied the depth-integrated model described above (see Equation (1)) to estimate the depth-integrated minimum and maximum fluorescence profiles (*F*_*d*_ and $F_{m,d}^{\prime }$) for 15 different biofilm thicknesses (range 0.01 mm to 4.51 mm) (see e.g., [8,9]) and 5 different downwelling attenuation coefficients (*k*_*d*_; range 0.07 mm^−1^ to 16.9 mm^−1^) (see e.g., [9,18,22,23]). Note that the maximum *k*_*d*_ modeled corresponds to the downwelling attenuation coefficient used in Serôdio [4]. For the upwelling fluorescence attenuation coefficient (*k*_*u*_), we scaled this relative to the downwelling attenuation coefficient (*k*_*d*_) in the manner of Serôdio [4] as *k*_*u*_ = *k*_*d*_(53.5/16.9).

To generate the expected depth-integrated fluorescence, we summed expected fluorescence from a series of 0.01 mm layers (i.e., increments), starting at *z* = 0 and ending with the total biofilm thickness being modeled (*z*_*max*_) (Equation (1)). First, surface irradiance was attenuated by exponential decay according to *z* and *k*_*d*_ for each 0.01 mm increment. This attenuated irradiance was then used to estimate the expected fluorescence at each depth *z* by linear interpolation (*approx* function in R) based on the depth-independent fluorescence profile (Figure S1). The expected at-depth fluorescence was then attenuated upward by *k*_*u*_. These profiles were then summed to obtain the fluorescence (*F*_*d*_ and $F_{m,d}^{\prime }$) expected to be measured at the surface for each modeled biofilm (15 × 5 = 75 total *F*_*d*_ and $F_{m,d}^{\prime }$ profiles per simulated community).

For modeling depth-integration effects on the two simulated heterogeneous communities, we modified Equation (1) to accommodate an HL-adapted top layer and an LL-adapted bottom layer:

(9) $$F_{d}\left(E,z,k_{d},k_{u}\right)=\int_{0}^{z_{\text{max}}/2}e^{-zk_{u}}F_{HL}\left(E,z,k_{d}\right)dz+\int_{z_{\text{max}}/2}^{z_{\text{max}}}e^{-zk_{u}}F_{LL}\left(E,z,k_{d}\right)dz$$

where *F*_*HL*_(*E*, *z*, *k*_*d*_) was the fluorescence profile for the HL-adapted upper layer (i.e., HL1 or HL2), and *F*_*LL*_(*E*, *z*, *k*_*d*_) was the fluorescence profile for the LL-adapted bottom layer (see profiles in Figure S1). (More specifically, because we summed expected fluorescence over a series of 0.01 mm layers, the upper layer was summed from *z* = 0 to *z* = (*z*_*max*_ − 0.01)/2 and the bottom layer was summed from *z* = (*z*_*max*_ + 0.01)/2 to *z* = *z*_*max*_.)

Estimates of *F*_*d*_ and $F_{m,d}^{\prime }$ over irradiance were combined to yield profiles of Φ_*PSII*_ (Equation (2)), then multiplied by *E* (the actual irradiance applied at the surface) to obtain *rETR*. These profiles were then used to estimate the photophysiological parameters for each thickness/attenuation combination using the hyperbolic tangent model of Jassby and Platt [19].

Bias. Recall that bias is the closeness to truth and here reflects the inherent method error. To assess predicted bias for the intact-biofilm method (depth-dependent), we simply compared the depth-integrated estimates of *ETR*_*m*_, *E*_*k*_ and *α* (based on Equations (1) and (9)) to the estimated true values (i.e., values in Table 1). We calculated both the absolute and relative error per parameter, for each simulated community and thickness/attenuation combination: absolute error = *estimate* − *true* and relative error = (*estimate* − *true*)/*true*.

For predicted bias in the slide method (depth-independent), as a first approximation, we assessed only the error expected from the fluorescence measurements on thin films (i.e., no light attenuation). For the homogeneous biofilms (LL, HL1 and HL2), the expected error in estimates was zero. For the heterogeneous biofilms (HL1LL and HL2LL), however, the photophysiological estimates were expected to have some error (see e.g., Equation (8)). To estimate this error, we first modeled the measured fluorescence yields as the simple average of the respective HL- and LL-profiles (Equations (6) and (7)). The average yields were then combined into the effective quantum yield Φ_*PSII*_ (Equation (8)), converted to *rETR* and then fit to the hyperbolic tangent model. We then calculated absolute and relative errors, as described for the depth-dependent estimates. (Note, the slide method could introduce greater bias through disproportionate subsampling of heterogeneous biofilms—this would be bias induced from measurement/sampling error as opposed to inherent method error. We simulate the possibility of sampling error in our estimations of precision.)

Precision. Recall that precision is the replicate variability and here reflects the repeat measurement error. To assess precision, we simulated the primary source of error for each technique. For the intact-biofilm method, it was assumed that the primary source of error would be spatial variability in biofilm thickness and resultant depth-integration effects on the fluorescence yields. For the slide method, it was assumed that the primary source of error would come from subsampling the biofilm. To estimate expected precision in the intact-biofilm method, we simulated replicate measurements for each biofilm community (i.e., LL, HL1, HL2, HL1LL, and HL2LL), for each thickness/attenuation combination. We simulated a random sample of *n* = 500, with a mean thickness equal to the modeled thickness (i.e., range 0.01 mm to 4.51 mm) and a standard deviation equal to 40% of the mean thickness (see e.g., [24]). The random samples were generated using the *rnorm* function in R. For simplicity, we assumed that *k*_*d*_ (and *k*_*u*_) were constant for each modeled biofilm and that only the thickness varied. For each random sample, we applied the appropriate depth-integration model to estimate the fluorescence yields (i.e., Equation (1) or (9)). The yields were then combined into the effective quantum yield Φ_*PSII*_, converted to *rETR*, and then fit to the hyperbolic tangent model. The standard deviation (SD) and coefficient of variation (CV) for each parameter were then calculated per sample.

For the slide method, we assumed maximum precision for the homogeneous biofilms (i.e., SD = 0 and CV = 0) because there would be no error associated with subsampling a truly homogeneous biofilm. For the two heterogeneous biofilms, however, subsampling was expected to introduce error in terms of how well the component proportions in the subsample matched the component proportions in the biofilm. We estimated maximum expected subsampling error based on a sample comprised of *n* = 3 subsamples: (1) equal proportions of the HL- and LL-layers; (2) all HL; and (3) all LL. The SD and CV were then calculated using parameter estimates assuming the slide method (see “Bias” subsection above), a homogeneous HL thin film and a homogeneous LL thin film (see also Table 1).

### 2.2. Empirical Evaluation of Bias and Precision

#### 2.2.1. Data Collection

Periphytic biofilms growing in stream mesocosms at the Environmental Protection Agency Experimental Stream Facility (ESF; Milford, OH, USA) were sampled in August 2018. The ESF contains sixteen 11-m stream mesocosms that are fed by natural river water from the East Fork of the Little Miami River (see Latham et al. [25] and Nietch et al. [26] for more detailed descriptions of this facility). The mesocosms were set up with a low light (LL) section upstream of a section with a higher light (HL) condition. The LL and HL conditions are meant to mimic approximately 10% and 100% of open canopy irradiance accomplished with full spectrum metal halide bulbs. Two types of substrate make up the streambed of the mesocosms: unglazed ceramic tiles and pieces of washed gravel. Flows over the tile tend to be more laminar compared to the turbulent flows produced by the gravel [27].

Nine tiles and two pieces of gravel were sampled from three control mesocosms. Sampling consisted of four replicate measurements on each intact biofilm by conventional PAM fluorometry. Following each replicate measurement, the area was subsampled for measurement by slide-based PAM fluorometry. This sampling protocol generated 43 pairs (one replicate pair was lost) of conventional and slide-based rapid light curves (RLCs).

By the time that the PAM fluorometry measurements were made on the mesocosm biofilms for this study, the biofilm structure had colonized and developed under continuous flow conditions over 72 d. The supplied water consisted of a constant delivery of natural river that was diluted and well-mixed with a continuous inflow of reverse osmosis water. This mixing created a nutrient profile more reflective of unimpacted streams in the Interior Plateau ecoregion, where the ESF resides and the native taxa used to produce the mesocosm communities come from. Mesocosm influent nitrogen (N) and phosphorus (P) content averaged 335, 284, and 12 μg L^−1^ of N for total N, nitrate-nitrite, and ammonium, respectively; and 43 and 33 μg L^−1^ of P for total P and total reactive P, respectively.

Table S1 includes measures characterizing relevant physical and chemical properties of the inhabiting periphyton, which had been determined from spatially randomized destructive sampling as part of a seasonal experimental schedule two days earlier. Periphytic biomass was about four times greater in the HL section compared to the LL section, both in terms of total dry weight and extracted chlorophyll content. The same pattern was observed in chlorophyll measurements by BenthoTorch fluorometry.

Fluorometric probe measures made with a BBE Moldaenke BenthoTorch on tile and gravel locations prior to destructive sampling suggested the relative abundance of diatoms, green algae, and cyanobacteria in the surficial biofilm was generally similar in the HL sections, while in the LL sections, it was comprised mostly of diatoms and cyanobacteria (Table S2). The prevalence of diatoms and cyanobacteria in LL sections was supported by microscope-based cell counts conducted on aliquots of periphyton slurries processed from destructive sampling immediately after the BenthoTorch measurements. The microscope-based cell counts, however, suggested that the mixture of cell densities was mostly cyanobacteria and green algae in the HL section, with few diatoms. Generally, the biofilms in the LL and HL sections were significantly different in terms of mass and with heterogeneous community structures and taxa dominance both between and within light environments and across replicate mesocosms (Tables S1–S3).

Conventional PAM. In the conventional PAM methodology, RLCs were obtained on intact biofilms using a Diving PAM II fluorometer (Heinz Walz GmbH, Effeltrich, Germany). For each RLC, the fiber optic probe of the Diving PAM II was placed in a plastic holder at a constant distance of 4 mm above the biofilm. Biofilms were not dark adapted before commencement of the RLC, but the holder excluded ambient light during measurement. Fluorescence yields were measured following a series of nine increasing pulses of irradiance (0 μmol m^−2^ s^−1^ up to a maximum of 593 μmol m^−2^ s^−1^) separated by 20-s acclimation intervals. Actual biofilm thickness was not quantified, but visual estimates suggested 0.6 mm to 1 mm under LL conditions and approximately 2 mm under HL conditions.

Slide-based PAM. After conducting an RLC on the intact biofilm, we removed the fiber optics and sample holder of the PAM and, using square-tipped forceps and a flat miniature spatula, scraped the entire biofilm under the fiber optics (~16 mm^2^ area). This subsample was placed on a glass cover slip and laminated as thinly (<0.2 mm thick) and evenly as possible on a microscope slide. We took care that the cover slip was pressed flat on the slide. The ensemble was inserted between the plastic leaf clip of the PAM fluorometer and the fiber optic cable was firmly clamped 4 mm above the sample. RLCs were then generated on an area of the laminated sample that had a minimum fluorescence (*F*_0_) of approximately 300 fluorescence units in a manner similar to the conventional PAM.

#### 2.2.2. Statistical Analysis

To assess bias, we compared estimates of *ETR*_*m*_, *E*_*k*_ and *α* for *n* = 43 paired intact-biofilm and slide measurements by paired t-test (*t.test* function in R). To assess precision, we first calculated SD and CV for each set of *n* = 4 replicate intact-biofilm or slide measurements per each of the 11 samples (*n* = 3 for one sample because one replicate pair was lost). We then compared the estimates of SD for *ETR*_*m*_, *E*_*k*_ and *α* between the intact-biofilm and slide methods using paired t-tests. We focused our t-test comparisons on SD rather than CV, because we assumed that the slide and intact-biofilm methods were both estimating the same true parameter per sample; we used estimates of CV for more general comparisons of precision across parameters because of the wide variation in parameter magnitudes. For t-test comparisons, we assumed a threshold of *p*_*i*_ ≤ 0.05/3 = 0.02 for statistical significance with Bonferroni correction for testing *m* = 3 parameters. We used the uncorrected threshold of *p*_*i*_ ≤ 0.05 to indicate statistical trends. All simulations, statistics, and figure generation were performed in R v. 3.6.2 [28].

## 3. Results

### 3.1. Theoretical Evaluation of Bias and Precision

#### 3.1.1. Numerical Simulations: Bias

**Figure 1.**
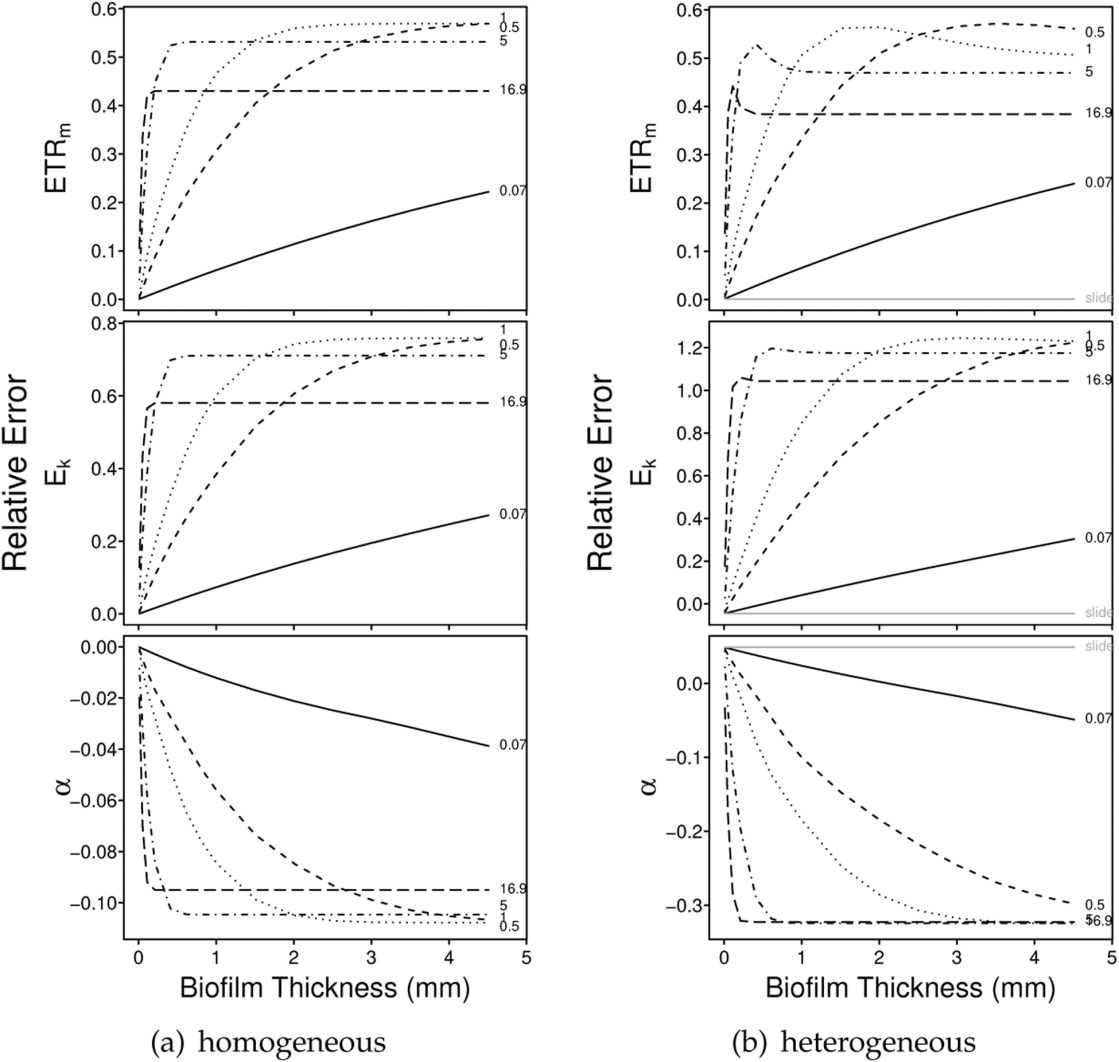
Relative error in estimates of *ETR*_*m*_, *E*_*k*_ and *α* for (**a**) a photophysiologically homogeneous biofilm (LL) of varying thickness and light attenuation; and (**b**) a photophysiologically heterogeneous biofilm (HL1LL) of varying thickness and light attenuation (see also Figure S3 for the HL1, HL2 and HL2LL communities). True values for each parameter can be found in Table 1. Each community was simulated over 15 biofilm thicknesses (range 0.01 mm to 4.51 mm) and 5 downwelling attenuation coefficients (*k*_*d*_: 0.07 mm^−1^, solid; 0.5 mm^−1^, dashed; 1 mm^−1^, dotted; 5 mm^−1^, dotdash; and 16.9 mm^−1^, longdash). For the homogeneous communities (e.g., (**a**)), the slide method is assumed to be completely unbiased. For the heterogeneous communities (e.g., (**b**)), however, the averaging of the fluorescence yields from the upper HL and lower LL layers results in slight bias in the slide-based estimates (indicated in (**b**), solid gray).

**Figure 2.**
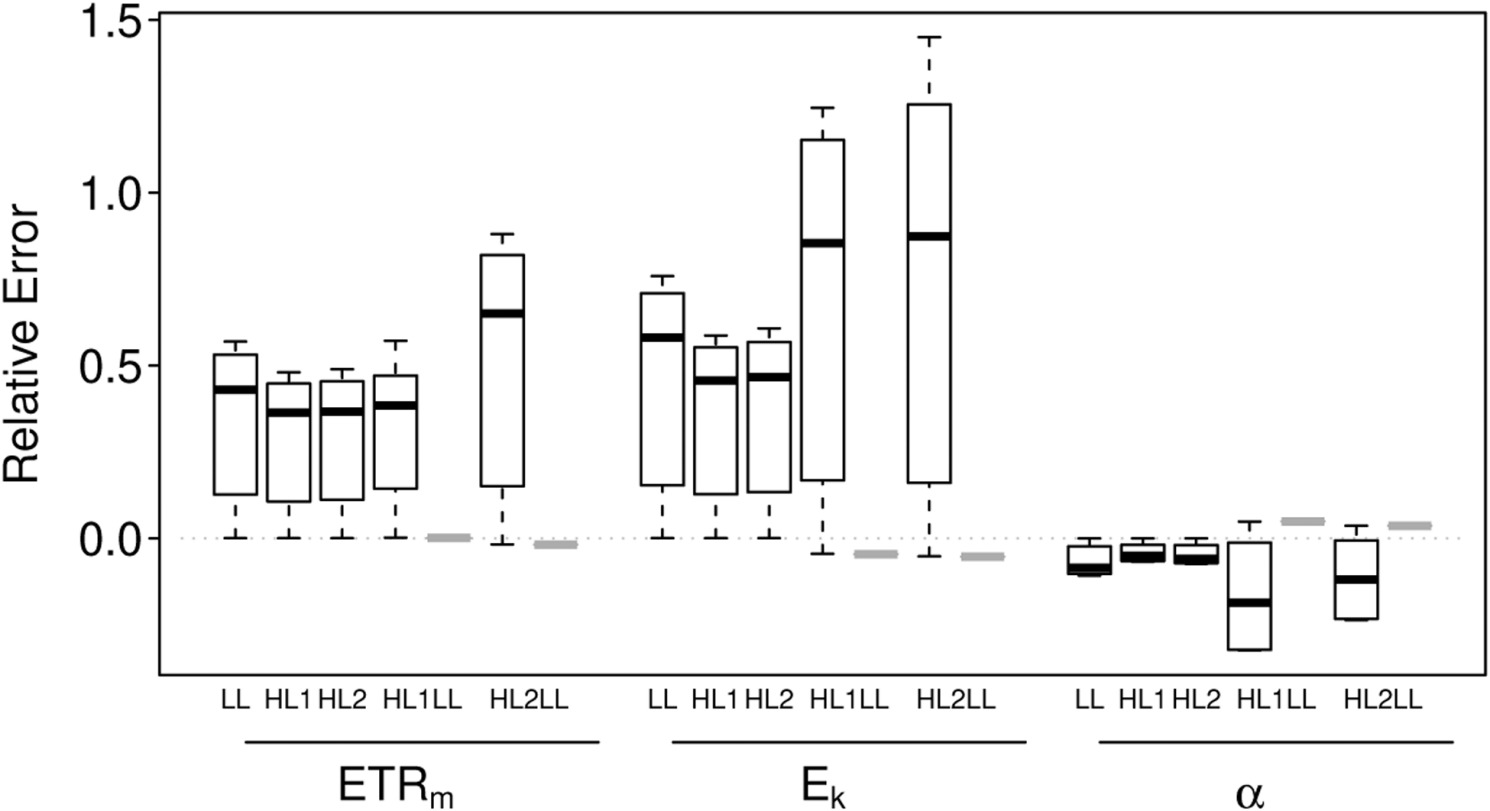
Boxplots of relative error for estimates of *ETR*_*m*_, *E*_*k*_ and *α* in the three photosynthetically homogeneous biofilms (LL, HL1, HL2) and the two photosynthetically heterogeneous biofilms (HL1LL, HL2LL). For HL1LL and HL2LL, bias predicted for the intact-biofilm method is indicated by the first boxplot (outlined in black); the second boxplot (outlined in gray and here just a thick bar) indicates the bias predicted for the slide method. The box-and-whiskers indicate the medians (central bar), first and third quartiles (box boundaries), and lower and upper extremes (“whiskers”) for each group; outliers are plotted as open circles.

For the photosynthetically homogeneous biofilms and based on relative errors, we observed an overestimation of *ETR*_*m*_ and *E*_*k*_ and slight underestimation of *α* with depth-integration effects, particularly as biofilm thickness increased (Figure 1a and Figure S3a,b). These biases tended to be exaggerated for the heterogeneous biofilms, particularly for *E*_*k*_ and *α* (Figure 1b and Figure S3c). For the depth-dependent intact-biofilm method, bias was predicted to be highest for estimates of *E*_*k*_ in heterogeneous communities and lowest for estimates of *α* in homogeneous communities (Figure 2). Mean relative errors predicted for *ETR*_*m*_, *E*_*k*_ and *α* were 0.30, 0.37, and −0.05 across the homogeneous biofilms; and 0.40, 0.71, and −0.14 across the heterogeneous biofilms.

In contrast, the depth-independent slide method was predicted to have no bias for the homogeneous biofilms (see subsection Bias in Section 2.1.3) and only slight bias for the heterogeneous biofilms (assuming representative subsampling of the biofilm). For the slide-based depth-independent measurements of heterogeneous biofilms, our simulations predicted that *ETR*_*m*_ would be relatively unbiased (mean relative error of −0.0086, 2% of the mean predicted relative error for depth-dependent measurements), while *E*_*k*_ would be slightly underestimated and *α* slightly overrestimated (*E*_*k*_, mean relative error of −0.049, 7% of the mean predicted relative error for depth-dependent measurements; and *α*, mean relative error of 0.043, 30% of the mean predicted relative error for depth-dependent measurements).

**Figure 3.**
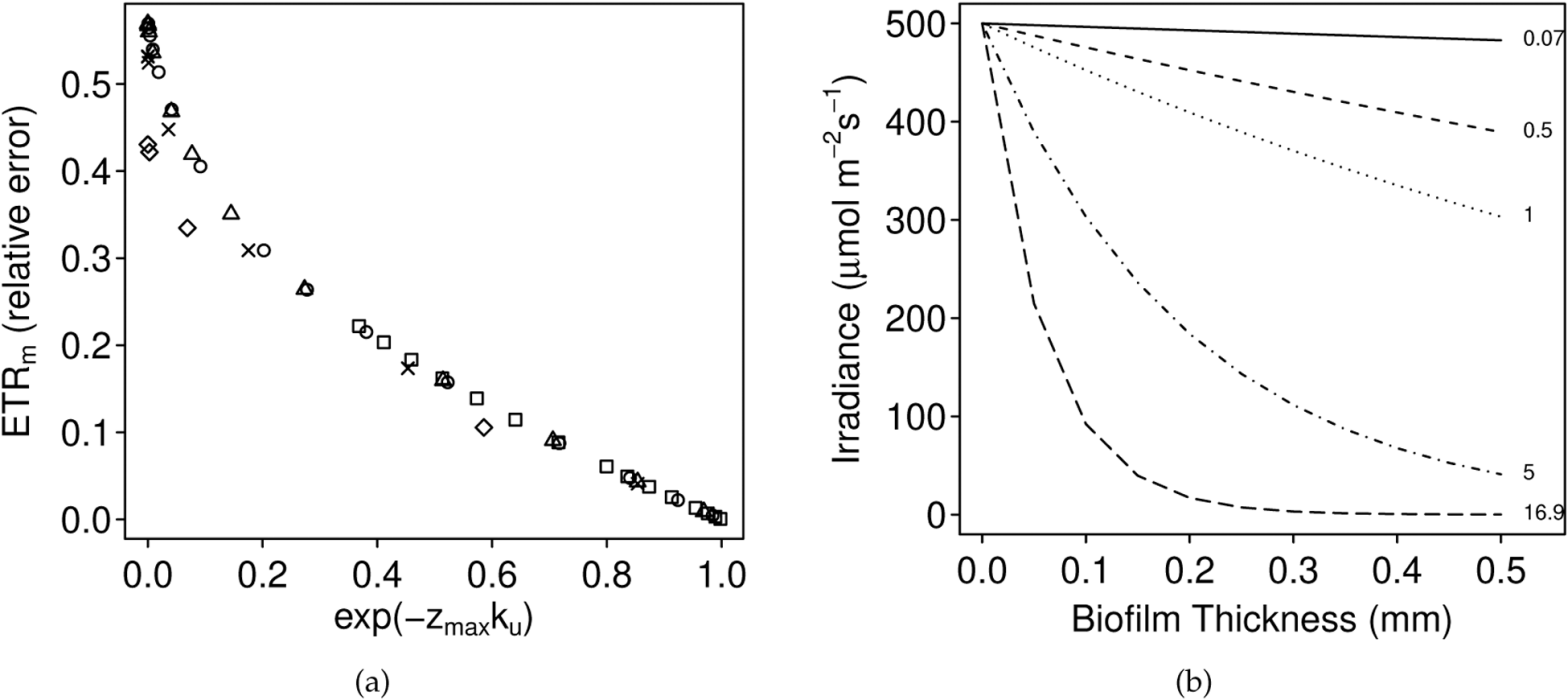
In (**a**), over/underestimation of the photophysiological parameters depends on both biofilm thickness (*z*_*max*_) and the attenuation coefficients (*k*_*d*_ and *k*_*u*_). Here, the relative error in *ETR*_*m*_ (i.e., depth-integrated estimate relative to the true depth-independent value) is plotted against the exponential of $-z_{\text{max}}k_{u}$. (Plot symbols indicate the downwelling attenuation coefficient (*k*_*d*_): 0.07 mm^−1^, squares; 0.5 mm^−1^, circles; 1 mm^−1^, triangles; 5 mm^−1^, crosses; and 16.9 mm^−1^, diamonds. The upwelling coefficient *k*_*u*_ was scaled relative to *k*_*d*_ following Serôdio [4]: *k*_*u*_ = *k*_*d*_(53.5/16.9). The relationship was stronger for *k*_*u*_, as opposed to *k*_*d*_, hence *k*_*u*_ is plotted here.) As the product of thickness and attenuation becomes large, the exponent becomes small and the relative error becomes large. In (**b**), the exponential decay of a surface irradiance of 500 μmol m^*−*2^ s^*−*1^ through a 0.5 mm biofilm for various values of *k*_*d*_. The steepness of the decay increases with the attenuation coefficient *k*_*d*_. For each *k*_*d*_, we can consider a critical depth or effective thickness (*z*_0_), at which the surface irradiance is essentially 0. For *k*_*d*_ = 16.9 mm^*−*1^, for example, the critical depth is approximately 0.13 mm. This would also be the effective biofilm thickness, i.e., the depth of the biofilm actually illuminated and photosynthetically activated.

In the depth-dependent intact-biofilm simulations, for each modeled downwelling attenuation coefficient, the magnitude of the bias increased with thickness until some maximum value was reached. The exact relationship was *k*-dependent (see e.g., Figure 3a) and maximum bias was reached at a critical depth, or depth zero (*z*_0_), below which the surface irradiance decayed to approximately 0 (see Figure S4). Beyond this critical depth, the predicted over/underestimation was asymptotic (i.e., beyond a certain thickness, the photophysiological estimates did not change). For thinner biofilms (e.g., <0.14 mm thick, *z*_0_ for *k*_*d*_ = 16.9 mm^*−*1^; Figure 1), the magnitude of the bias increased with *k*_*d*_. For thicker biofilms (e.g., >33 mm thick, *z*_0_ for *k*_*d*_ = 0.07 mm^−1^; Figure 1), the magnitude of the bias decreased with *k*_*d*_.

The predicted maximum bias was a function of the fluorescence profiles of the simulated biofilm and the downwelling attenuation coefficient. Specifically, as *k*_*d*_ increased, the magnitude of the maximum bias decreased (Figure S5). These outcomes followed from the exponential decay of surface irradiance through a biofilm (see e.g., Figure 3b): as *k*_*d*_ became large, the irradiance decayed more rapidly, such that lower layers of the biofilm were no longer illuminated and activated. For example, in Figure 3b, when the attenuation coefficient was very large (e.g., 16.9 mm^−1^), the irradiance decayed to near 0 within the top 0.2 mm, such that estimates of *ETR*_*m*_, *E*_*k*_ and *α* did not change substantially for biofilms thicker than 0.2 mm (for *k*_*d*_ = 16.9 mm^−1^; Figure 1). We can also consider the effective biofilm thickness for a given *k*_*d*_—this thickness is the same as *z*_0_ and reflects the depth of the biofilm that is actually illuminated and photosynthetically activated (see Figures S4 and S6).

#### 3.1.2. Numerical Simulations: Precision

**Figure 4.**
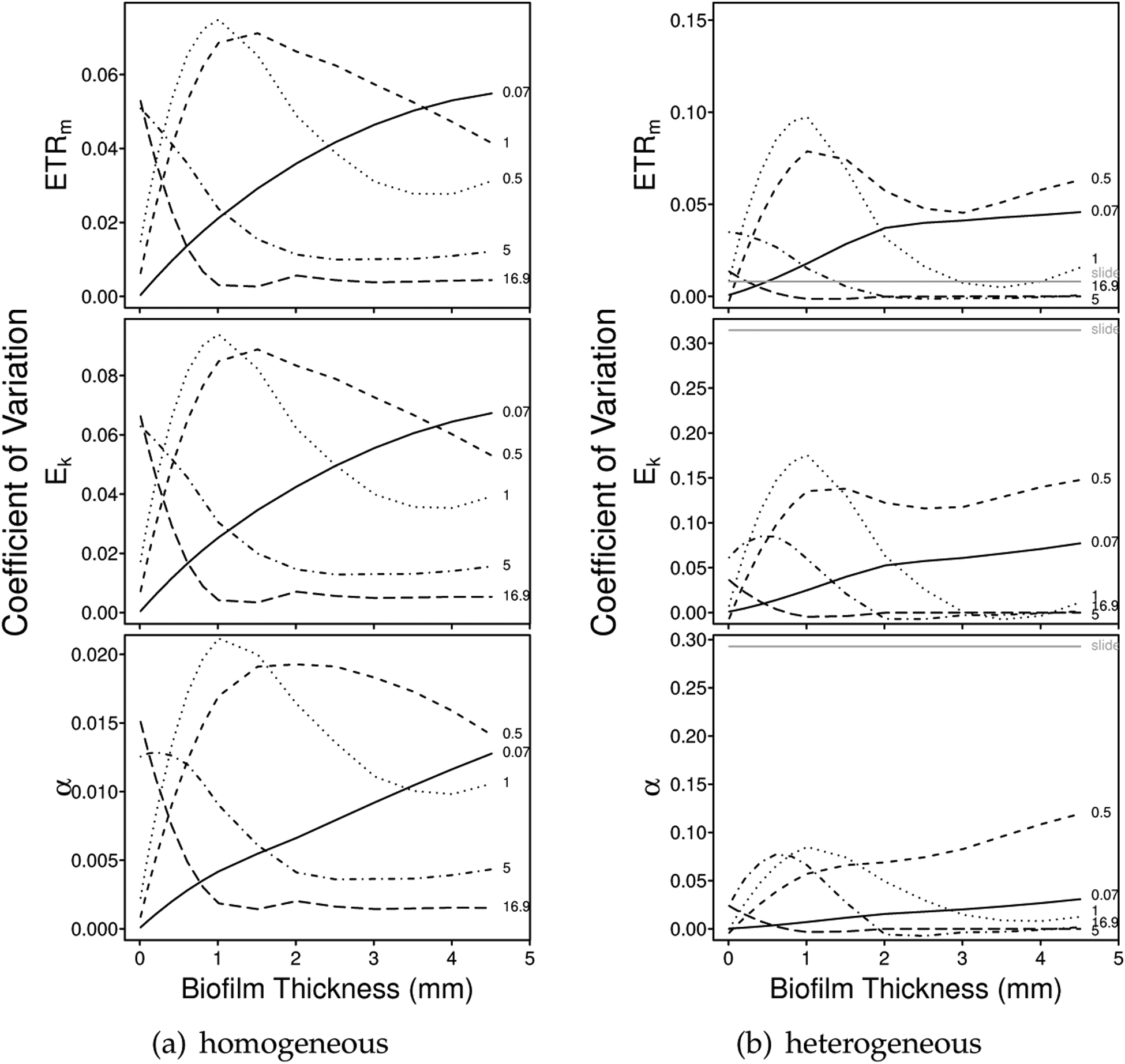
Coefficient of variation for estimates of *ETR*_*m*_, *E*_*k*_ and *α* in (**a**) a photophysiologically homogeneous biofilm (LL) of varying thickness and light attenuation; and (**b**) a photophysiologically heterogeneous biofilm (HL1LL) of varying thickness and light attenuation (see also Figure S7 for the HL1, HL2 and HL2LL communities). Each community was simulated over 15 biofilm thicknesses (range 0.01 mm to 4.51 mm) and 5 downwelling attenuation coefficients (*k*_*d*_: 0.07 mm^*−*1^, solid; 0.5 mm^*−*1^, dashed; 1 mm^*−*1^, dotted; 5 mm^*−*1^, dotdash; and 16.9 mm^−1^, longdash). For the homogeneous communities (e.g., (**a**)) the slide method is assumed to have a CV of 0. For the heterogeneous communities (e.g., (**b**)), however, improper subsampling results in imprecision (indicated in (**b**), solid gray). (Note, the lines plotted were smoothed by LOESS using the *loess* function in R.)

Our simulations of the depth-dependent intact-biofilm method suggested that precision varied with biofilm thickness and attenuation (Figure 4 and Figure S7). More specifically, for a given attenuation coefficient *k*_*d*_, the CV first increased and then decreased as the biofilm thickened. Typically, the maximum CV occurred around 0.33 × *z*_0_ (not shown). Across *k*_*d*_, mean precision tended to be higher for very small attenuation coefficients (e.g., *k*_*d*_ = 0.07 mm^−1^)—where the rate-of-change in parameter bias was slower with thickness (e.g., compare initial regions of the curves in Figure 1 and Figure S3)—and for very large attenuation coefficients (e.g., *k*_*d*_ 5 mm^−1^)—where the rapid attenuation of light resulted in only the uppermost layers of the biofilm being activated and contributing to the depth integration effects (see e.g., Figure S8).

**Figure 5.**
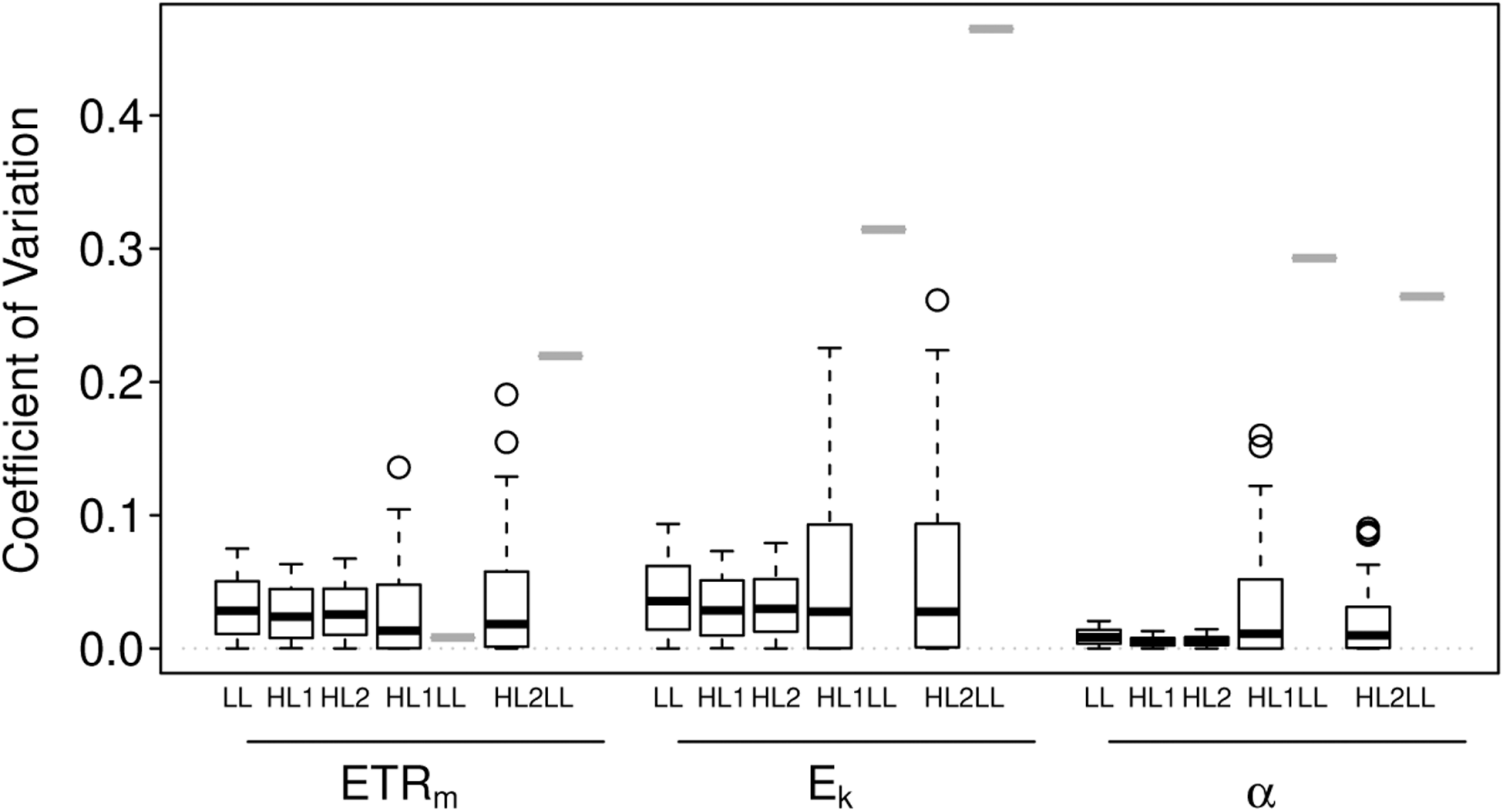
Boxplots of the coefficient of variation (CV) for estimates of *ETR*_*m*_, *E*_*k*_ and *α* in the three photosynthetically homogeneous biofilms (LL, HL1, HL2) and the two photosynthetically heterogeneous biofilms (HL1LL, HL2LL). For HL1LL and HL2LL, precision predicted for the intact-biofilm method is indicated by the first boxplot (outlined in black); the second boxplot (outlined in gray and here just a thick bar) indicates the maximum CV (minimum precision) predicted for the slide method. The box-and-whiskers indicate the medians (central bar), first and third quartiles (box boundaries), and lower and upper extremes (“whiskers”) for each group; outliers are plotted as open circles.

Precision tended to be highest for the estimates of *α*, particularly for the homogeneous biofilms, and lowest for the estimates of *E*_*k*_ (Figure 5). For the homogeneous biofilms, mean CVs were 0.029, 0.035 and 0.007 for *ETR*_*m*_, *E*_*k*_ and *α*, respectively; and for the heterogeneous biofilms, mean CVs were 0.029, 0.049 and 0.023 for *ETR*_*m*_, *E*_*k*_ and *α*, respectively.

The maximum predicted CV tended to be highest for the depth-independent slide method (Figure 5). This pattern was particularly true for the estimates of *E*_*k*_ and *α*—very poor subsampling of the simulated heterogeneous biofilms resulted in CVs up to 0.22 for *ETR*_*m*_, 0.46 for *E*_*k*_, and 0.29 for *α* (1.5×, 1.8× and 1.6× maximum predicted CVs in the depth-dependent simulations).

### 3.2. Empirical Evaluation of Bias and Precision

#### 3.2.1. Intact-Biofilm-Method vs. Slide-Method: Bias

**Figure 6.**
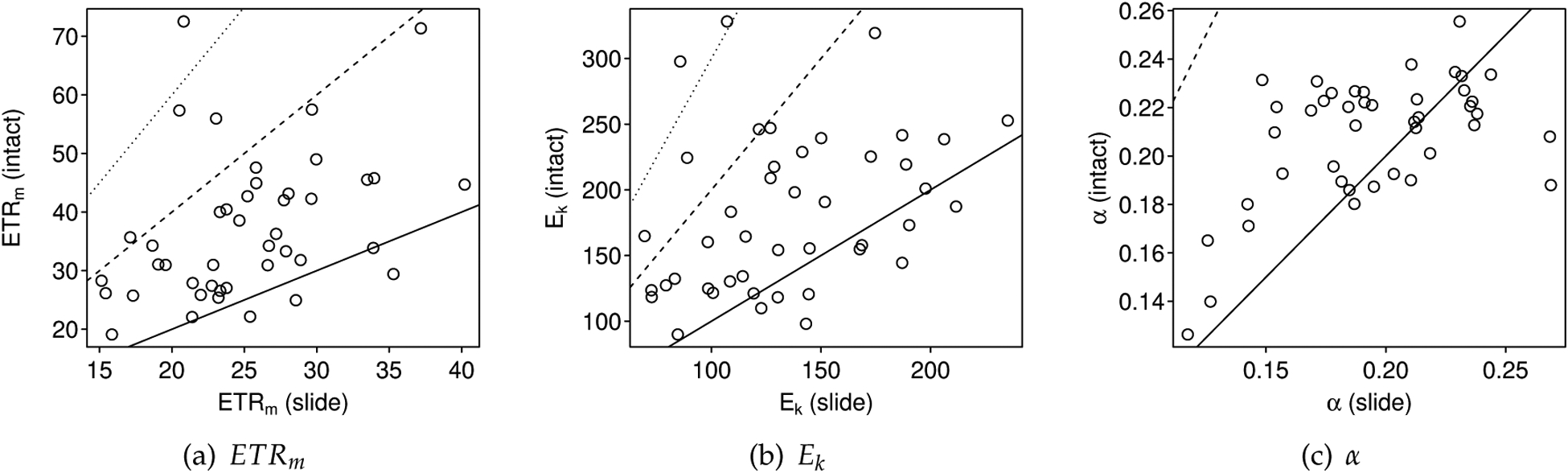
Comparisons of (**a**) *ETR*_*m*_, (**b**) *E*_*k*_ and (**c**) *α* as estimated using the conventional intact-biofilm method (i.e., depth-dependent) and the novel slide method (i.e., depth-independent). In each subfigure, the intact-biofilm-based estimate is plotted relative to its paired slide-based estimate. The solid black line indicates the 1:1 relationship: points falling above and to the left of the line are overestimated by the intact-biofilm method and points falling below and to the right of the line are underestimated by the intact-biofilm method (relative to the slide method). Relative error (RE) for the intact-biofilm-based estimates are also indicated assuming the slide-based estimates to be true: the dashed black line indicates RE = 1 and the dotted black line indicates RE = 2.

Parameter estimates were compared between paired intact-biofilm (depth-dependent) and slide-based (depth-independent) measurements (Figure 6). Intact-biofilm-based estimates of *ETR*_*m*_ were greater than slide-based estimates of *ETR*_*m*_ for 39 out of 43 paired samples (91%, *t*_42_ = 6.90, *p* < 0.001; Figure 6a). For *E*_*k*_, intact-biofilm-based estimates were greater than slide-based estimates for 31 out of 43 paired samples (79%, *t*_42_ = 5.06, *p* < 0.001; Figure 6b). For *α*, intact-biofilm-based estimates were less than slide-based estimates for 14 out of 43 paired samples (33%, *t*_42_ =−2.73, *p* < 0.009; Figure 6c). If we assume the slide-based estimates to be true, then mean relative errors in the intact-biofilm-based estimates are 0.52 (range −0.17 to 2.48) for *ETR*_*m*_; 0.43 (range −0.32 to 2.47) for *E*_*k*_; and 0.09 (range −0.30 to 0.56) for *α*.

#### 3.2.2. Intact-Biofilm-Method vs. Slide-Method: Precision

**Figure 7.**
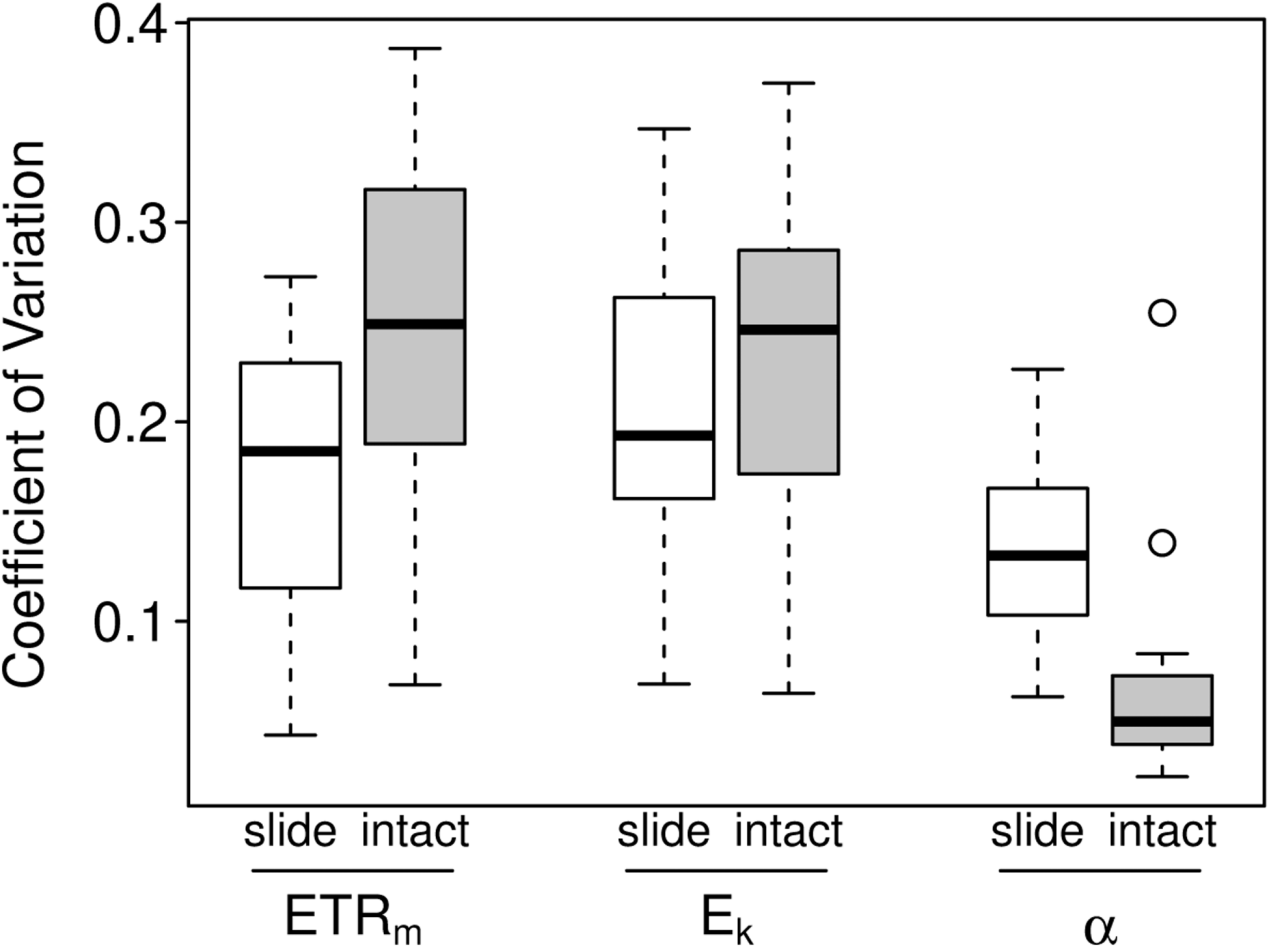
Boxplots of the coefficient of variation in estimates of *ETR*_*m*_, *E*_*k*_ and *α* for the slide (unshaded) and intact-biofilm (shaded) methods. The CV was estimated from *n* = 4 replicate measurements per method per sample (11 samples total). The box-and-whiskers indicate the medians (central bar), first and third quartiles (box boundaries), and lower and upper extremes (“whiskers”) for each group; outliers are plotted as open circles.

In comparisons of precision, the slide method was significantly more precise than the intact-biofilm method for *ETR*_*m*_ (SD comparison: *t*_10_ = 3.24, *p* = 0.009; Figure 7); whereas the intact-biofilm method tended to be more precise than the slide method in estimating *α* (SD comparison: *t*_10_ = −2.45, *p* = 0.034). Both methods were equally precise in estimating *E*_*k*_ (SD comparison: *t*_10_ = 1.66, *p* = 0.128). For both methods, precision was generally higher for estimates of *α* (mean CV = 0.11) than for estimates of *ETR*_*m*_ and *E*_*k*_ (mean CVs = 0.20 and 0.22; see also Figure 7).

## 4. Discussion

Serôdio [4] and others [5–7] demonstrated numerically and experimentally that fluorescence-based estimates of *ETR*_*m*_ and *E*_*k*_ tended to be overestimated (substantially) and *α* underestimated (minorly) for measurements conducted on intact sediment biofilms as opposed to suspended or thin-film algae. The inaccuracies in the intact samples were induced by depth integration effects resulting from vertical attenuation of both the measuring and actinic light and the fluorescence signal. Here, we extended the work of Serôdio [4] by exploring the impacts of depth integration on biofilms of varying thickness and composition (e.g., photophysiologically heterogeneous as opposed to homogeneous). The primary motivation was to make theoretical predictions of bias and precision for the conventional intact biofilm (depth-dependent) method and a novel slide-based (depth-independent) method. These theoretical predictions were supported by experimental observations from paired intact-biofilm and slide measurements.

Our numerical simulations of bias predicted that the intact-biofilm method would overestimate *ETR*_*m*_ and *E*_*k*_—and slightly underestimate *α*—because of depth-integration effects; whereas, the slide-based method should be relatively unbiased (assuming representative subsampling). Similarly, through empirical observation, we observed that *ETR*_*m*_ and *E*_*k*_ were consistently higher for the intact-biofilm-based measurements relative to paired slide-based measurements. Estimates of *α*, however, tended to be higher rather than lower for the intact-biofilm measurements. Our numerical simulations of precision suggested that the slide-based method should be more precise for homogeneous biofilms, but potentially less precise for heterogeneous biofilms because of the potential for subsampling error—particularly for *E*_*k*_ and *α*. More generally, these simulations predicted that precision (regardless of measurement technique) would be highest for estimates of *α* and lowest for estimates of *E*_*k*_. These predictions were somewhat matched by our empirical observations of precision. Specifically, we found precision to be highest for estimates of *α*—particularly when using the intact-biofilm method. Precision was typically lower for estimates of *ETR*_*m*_ and *E*_*k*_. For *ETR*_*m*_, however, the slide method demonstrated more precision than the intact-biofilm method.

Our study demonstrates that the technique of taking a vertically representative biofilm subsample and pressing it into a thin layer prior to fluorescence measurements (i.e., slide-based method) offers substantial reduction in error, albeit some potential loss in precision, in comparison to the conventional practice of taking fluorescence measurements directly on intact biofilms. Even fairly thin biofilms (e.g., <1 mm thick) are subject to relative errors up to 80% for *ETR*_*m*_ and 140% for *E*_*k*_, depending on *k*_*d*_, for measurements made on the intact biofilm. In contrast, the proposed slide-based method, though somewhat destructive, requires only a small subsample, is easy to implement in the field, and eliminates much of the error incurred from depth-integration effects on intact biofilms.

### 4.1. Bias

Several previous studies have reported the overestimation of key photophysiological parameters for photosynthesizing samples with depth or optical density [4–7]. Our simulations are consistent with these observations and provide additional insights into potential biases. Namely, our simulations demonstrate that (1) the magnitude of the bias is *k* and *z*-dependent and (2) the potential bias is even greater for photosynthetically heterogeneous samples. For the former, we observed that, for a given *k*_*d*_, the bias increased asymptotically with sample thickness. Bias was greatest in thick biofilms and had an inverse relationship with *k*_*d*_. For the latter, this is consistent with simulations by Serôdio [4] for different vertical distributions of microalgae in the sediment: when microalgae were concentrated near the surface, bias in *ETR*_*m*_ and *E*_*k*_ increased.

The slide-based technique was also subject to bias: from both the averaging of the fluorescence yields in heterogeneous samples (intrinsic) and from disproportionate subsampling. Error introduced from averaging effects is expected to be minimal (e.g., mean relative error for *ETR*_*m*_ was −0.0086 in the slide-based simulations, only 2% of the mean predicted relative error for the depth-dependent simulations). The potential for error because of poor subsampling, however, was more substantial, but still remained less than the potential for error induced by depth-integration effects (e.g., the maximum relative error predicted for *ETR*_*m*_ from poor subsampling was 0.25, whereas the maximum relative error predicted for *ETR*_*m*_ from depth-integration effects was 0.88).

With respect to bias, the slide-based technique outperforms the conventional method for samples with depth. The slide-based technique should particularly be preferred for samples that are suspected of being heterogeneous and are thick with the potential to have small attenuation coefficients. Our empirical data supported our simulations. Intact-biofilm-based estimates of *ETR*_*m*_ and *E*_*k*_ were higher than 91% and 79% of the paired slide based estimates. Furthermore, treating the slide-based estimate as true, intact-biofilm estimates of *ETR*_*m*_ and *E*_*k*_ had mean relative errors of 0.52 and 0.43.

### 4.2. Precision

To our knowledge, no previous studies have addressed the impacts of depth integration on precision of PAM-derived photophysiological measurements in microalgal biofilms. Our simulations suggested only modest effects of depth-integration on precision with mean CVs less than 0.1, and a maximum of 0.26 (for *E*_*k*_) across all simulations. Replicate variability tended to be highest for *k*_*d*_ greater than 0.7 mm^−1^ and less than 5 mm^−1^. Replicate variability was lower for very small *k*_*d*_ because the rate-of-change in parameter bias was slower; and lower for very large *k*_*d*_ because light was more rapidly attenuated and only the uppermost layers of the biofilm were becoming activated.

In contrast, the potential for subsampling error resulted in mean CVs greater than 0.1 and a maximum of 0.46 (also for *E*_*k*_) in our simulations of the slide-based technique. Nonetheless, our empirical observations suggested a slight improvement in precision for the slide-based technique. Observed precision was equivalent between the two techniques for *E*_*k*_ and significantly higher with the slide-technique for measurement of *ETR*_*m*_. Specifically, the CV for *ETR*_*m*_ was reduced by 30% when using the slide method in comparison to the intact-biofilm method. For *α*, the intact-biofilm method was more precise (e.g., CV for *α* was increased by 100% when using the slide method in comparison to the intact-biofilm method). Our observations suggest, however, that the interpretation of *α* is problematic.

### 4.3. The Problem with α

In terms of bias induced by depth integration, our simulated predictions were consistent with our empirical observations for estimates of *ETR*_*m*_ and *E*_*k*_. For example, our original simulations found that *ETR*_*m*_ and *E*_*k*_ were overestimated in 98% and 95% of the 75 simulations per modeled biofilm. Similarly, our empirical data resulted in higher estimates for *ETR*_*m*_ and *E*_*k*_ in 91% and 79% of 43 intact-biofilm-based measurements compared to paired slide-based measurements. We additionally applied our depth-integration simulation (i.e., 75 combinations of *k*_*d*_, ranging from 0.07 mm^*−*1^ to 16.9 mm^−1^, and biofilm thicknesses, ranging from 0.01 mm to 4.51 mm) to the 43 empirical slide-based fluorescence profiles. These simulations predicted a 100% overestimation of *ETR*_*m*_ and *E*_*k*_ from depth-integration effects on the “true” slide-based measurements.

In contrast, simulation and empirical observation were inconsistent for estimates of *α*. Our original simulations resulted in the underestimation of *α* for 90% of the modeled scenarios, whereas *α* was lower in only 33% of the slide-based measurements compared to paired intact-biofilm measurements. Further, when we applied the depth integration simulations to the empirical slide-based fluorescence profiles, *α* was underestimated for 77% of the modeled scenarios. For some of these empirical profiles, the predicted over/underestimation of *α* varied with *k*_*d*_ and biofilm thickness (Figure S9c).

**Figure 8.**
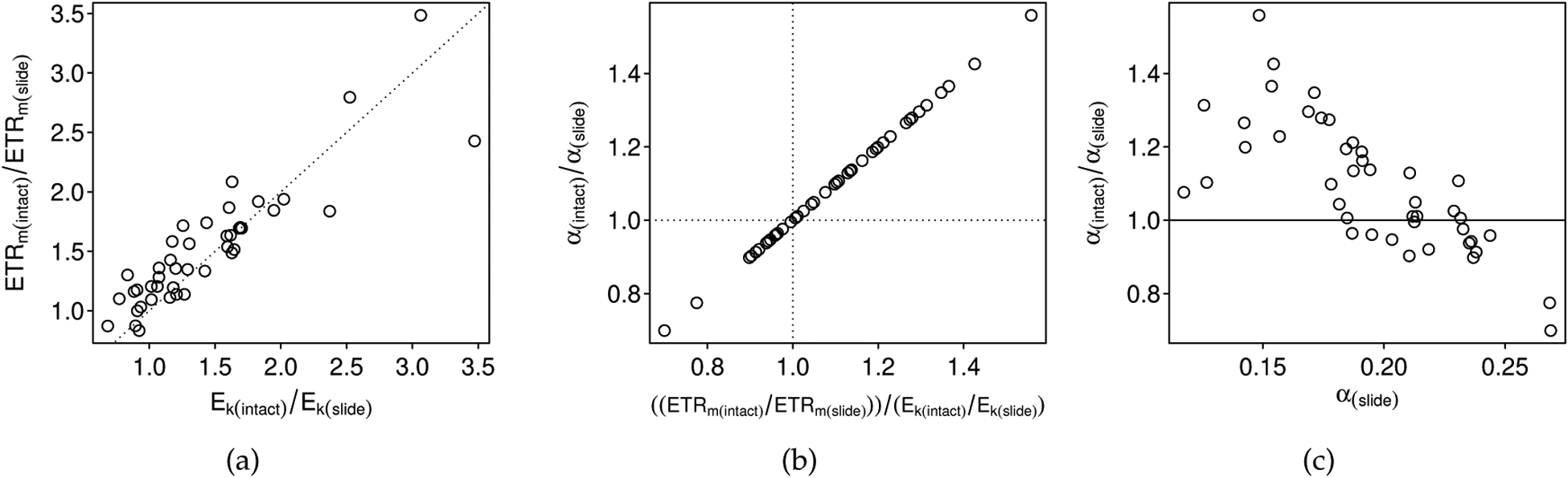
The over or underestimation of *α* with depth integration corresponds to the relative changes in *ETR*_*m*_ and *E*_*k*_ with depth integration. In (**a**), we plot the depth-dependent (intact-biofilm) estimate for *ETR*_*m*_ relative to its paired depth-independent (slide) estimate against the same for *E*_*k*_. The dotted line indicates the 1:1 change. Above and to the left of this line, *ETR*_*m*_ is overestimated more than *E*_*k*_ because of depth integration effects and *α* will therefore be overestimated. Below and to the right of the dotted line, *E*_*k*_ is overestimated more than *ETR*_*m*_ because of depth-integration effects and *α* will therefore be underestimated. In (**b**), we plot the depth-dependent (intact-biofilm) estimate for *α* relative to its paired depth-independent (slide) estimate against the ratio of the relative change in *ETR*_*m*_ to the relative change in *E*_*k*_. The horizontal and vertical dotted lines divide the figure into quadrants indicating over or underestimation: samples in the top right quadrant were observed to have higher estimates of *α* with the intact-biofilm method (as expected because *ETR*_*m*_ was overestimated more than *E*_*k*_); samples in the bottom left quadrant were observed to have lower estimates of *α* with the intact-biofilm method (as expected because *E*_*k*_ was overestimated more than *ETR*_*m*_). In (**c**), we plot the depth-dependent (intact-biofilm) estimate for *α* relative to its paired depth-independent (slide) estimate against the depth-independent (slide) estimate for *α*. The solid horizontal line indicates the 1:1 relationship: above this line, the estimate for *α* is higher in the intact-biofilm method, and below this line, the estimate for *α* is lower in the intact-biofilm method.

Because *α* is equivalent to *ETR*_*m*_/*E*_*k*_, whether *α* becomes over or underestimated with depth-integration depends on the relative change in *ETR*_*m*_ and *E*_*k*_: i.e., the ratio *R*_*ETR*_ = *ETR*_*m*,*d*_/*ETR*_*m*_ versus the ratio *R*_*Ek*_ = *Ek*_*m*,*d*_/*ETR*_*m*_. When *R*_*ETR*_/*R*_*Ek*_ = 1, *α* is unaffected by depth integration. When *R*_*ETR*_/*R*_*Ek*_ > 1 (i.e., *ETR*_*m*_ is overestimated more), then *α* will be overestimated. When *R*_*ETR*_/*R*_*Ek*_ < 1 (i.e., *E*_*k*_ is overestimated more), then *α* will be underestimated. For the empirical data, the comparison of *R*_*ETR*_ and *R*_*Ek*_ (e.g., where *R*_*ETR*_ = *ETR*_*m*(*intact–biofilm*)_/*ETR*_*m*(*slide*)_) accurately predicted whether *α* would be higher or lower in the intact-biofilm-based measurements compared to the slide-based measurements (Figure 8a,b). There appeared to be some predictability in the direction of bias for *α*. A comparison of *R*_*α*_ = *α*_(*intact–biofilm*_)/*α*_(*slide*)_ to the “true” depth-independent values (i.e., slide-based) from our empirical observations revealed a significant negative relationship (*r* = −0.76, *p* < 0.001, Figure 8c; although note, that the correlation was weaker, *r* = −0.59, if the simulated data were also considered (not shown)).

The parameter *α* represents the maximal increase in *rETR* per change in irradiance and provides a measure of photosynthetic efficiency [1,3,29]. When *α* is large, *rETR* increases rapidly at low irradiance—a characteristic often observed in low-light adapted plants and algae, which tend to optimize light harvesting capacity at lower irradiances (e.g., [3,30,31]). The initial RLC slope or *α* is also one of two key parameters constraining photosynthesis: (1) the ability to increase photosynthesis when light is the limiting factor (i.e., *α*) and the maximal capacity to photosynthesize when light is unlimited (*ETR*_*m*_) (e.g., [29]). The demonstrable variability in *α* in both simulations and observations, however, warrants caution, both in its interpretation and use for estimating primary productivity under light-limiting conditions.

Our observations suggest that this parameter may be particularly unreliable for samples with depth because it can be sometimes overestimated and sometimes underestimated—primarily depending on the relative bias in *ETR*_*m*_ and *E*_*k*_. Others have urged caution as well in interpreting *α*. Both Jassby and Platt [19] and Ralph and Gademann [3], for example, observed that *α* was susceptible to underestimation if the sampling frequency was too low in the light-limiting region of P–E and rETR–E curves. Estimation of *α* appears also to be particularly susceptible to choice of model for curve fitting [19,32,33]. As an additional check, we estimated *α* by simple linear regression (*lm* function in R) over the linear portion of the rETR–E curve (specifically, we used the first four data points, with typical maximum irradiance of 95 μmol m^−2^ s^−1^ or less). We obtained the linear estimates of *α* on all 86 empirical RLCs and all simulated (depth-integrated) RLCs. The two model estimates of *α* were strongly correlated (*r* = 0.97, *p* < 0.001). The hyperbolic tangent model typically yielded a higher estimate of *α* (mean relative error of 9% with respect to the linear model; Figure S10a). In comparisons of bias (i.e., depth-dependent estimates relative to depth-independent estimates, across all empirical and simulated data) between the two model estimates of *α*, the models were again highly correlated (*r* = 0.79, *p* < 0.001), albeit with the hyperbolic tangent model tending to underestimate and the linear model tending to overestimate the “true” slope (mean relative errors of −0.024 and 0.045; Figure S10b).

## 5. Conclusions

We recommend the slide method as a technique to minimize depth integration effects and reduce bias in estimates of key photophysiological parameters when using PAM fluorometry. While there are currently other techniques to compensate for depth integration error [4,6,15,16], many of these require complicated setups and can only be performed in the lab. In contrast, the slide method is relatively simple and easily implemented, even in the field. This technique additionally offers increased precision in the estimate of *ETR*_*m*_. A caveat, however, is that subsampling errors can result in imprecision for *E*_*k*_ and *α* and introduce bias. Given the risk of subsampling error, it is worth considering the character of the substrate to be sampled—substrates that are heterogeneous or thick with small attenuation coefficients are more prone to larger depth-integration error, and thus, are particularly recommended for the application of the new, slide-based technique presented here for PAM fluorometry.

## Supplementary Material

Supplement1

## Figures and Tables

**Table 1.** Photophysiological parameter estimates for the synthesized light-adapted algae. We simulated three photosynthetically homogeneous biofilms (LL, HL1, HL2) and two heterogeneous biofilms (HL1LL, HL2LL) (see text for details).

	*ETR*_*m*_	*E*_*k*_	*α*
LL	100.6	164	0.61
HL1	100.5	301	0.33
HL2	155.3	428	0.36
HL1LL	99.0	215	0.46
HL2LL	123.9	281	0.44

## Abbreviations

The following abbreviations are used in this manuscript:

*α*: Photosynthetic efficiency
CV: Coefficient of variation
*E*: Surface irradiance
*E_k_*: Minimum saturating irradiance
ESF: USEPA Experimental Stream Facility
*ETR_m_*: Maximum electron transport rate
*F*: Minimum fluorescence yield
$F_{m}^{\prime }$: Maximum fluorescence yield
HL: High light
*k_d_*: Downwelling attenuation coefficient
*k_u_*: Upwelling attenuation coefficient
LL: Low light
PAM: Pulse-amplitude modulated
P–E: Photosynthesis–irradiance
Φ_*II*_: Effective quantum yield
rETR: Relative electron transport rate
RLC: Rapid light curve
SD: Standard deviation
*z*_0_: Critical depth or effective biofilm thickness
*z_max_*: Total thickness of biofilm
