# Supplementary material for: A Novel Thin-Film Technique to Improve Accuracy of Fluorescence-Based Estimates for Periphytic Biofilms
Source: Water (Basel). Author manuscript; Available in PMC 2022 May 23. (PMC8262624; doi:10.3390/w13111464)
Supplement: Supplement1 [file NIHMS1713115-supplement-Supplement1.zip › water-1187386-supple.pdf]

Supplementary Materials: A novel thin-film technique to improve accuracy of fluorescence-based estimates for periphytic biofilms

Leon Katona [1], Yvonne Vadeboncoeur [1], Christopher T. Nietch [2] and Katie Hossler [1]*

1. Supplementary Tables

Table S1. Representative physical and chemical properties of the biofilms assessed by PAM fluorometry. Bulk periphyton measurements were made on aliquots of slurries prepared from destructive sampling of gravel and tiles. Ten pieces of gravel and two tiles were sampled from each mesocosm. The periphyton scraped and rinsed from the substrates was pooled into a single slurry. For each mesocosm and light condition the total dry weight (Dry Wt, mg cm^{-2}), ash-free dry mass (AFDM, mg cm^{-2}), organic matter (OM, %), extracted chlorophyll-*a* ($ChlA_{ex}$, µg cm^{-2}; phaeophytin-corrected) and BBE BenthoTorch estimate of chlorophyll-*a* ($ChlA_{bt}$, µg cm^{-2} are indicated for the representative biofilms.(The PAM fluorometry measurements presented in the manuscript were taken two days later on mesocosm biofilms that had not been destructively sampled. Note also, that high light biofilm substrates included both gravel and tile for the PAM fluorometry.)

Mesocosm	Substrate	Lighting	Dry Wt	AFDM	OM	$ChlA_{ex}$	$ChlA_{bt}$
E01.1	Tile	Low	1.10	0.80	73.2	4.85	2.84
E03.2	Tile	Low	3.94	1.29	32.6	10.80	2.98
E07.1	Tile	Low	1.25	0.46	37.1	3.82	2.94
E01.1	Gravel	High	11.61	4.59	39.5	51.92	6.89
E03.2	Gravel	High	12.39	4.28	34.6	21.93	4.33
E07.1	Gravel	High	5.13	2.34	45.6	14.26	4.90

Table S2. Representative taxa compositions of the biofilms assessed by PAM fluorometry. Relative abundances (%) of Cyanobacteria (Cyano), Chlorophyta (Green; i.e. green algae), and Bacillariophyta (Diatom) are indicated based on either BBE BenthoTorch fluorometry or microscope cell counts. BBE BenthoTorch measurements were made on four pieces of gravel (high light condition) and two tiles (low light condition) for each mesocosm. Microscope-based cell counts were made on on aliquots of periphyton slurries processed from destructive sampling of ten pieces of gravel (high light) and two tiles (low light) per mesocosm. (The PAM fluorometry measurements presented in the manuscript were taken two days later on mesocosm biofilms that had not been destructively sampled. Note also, that high light biofilm substrates included both gravel and tile for the PAM fluorometry.)

		BBE BenthoTorch			Microscope Cell Counts		
**Mesocosm**	**Lighting**	**Cyano**	**Green**	**Diatom**	**Cyano**	**Green**	**Diatom**
E01.1	Low	43	8	49	36	35	28
E03.2	Low	41	0	59	49	3	48
E07.1	Low	41	0	59	38	18	44
E01.1	High	21	28	51	8	69	23
E03.2	High	47	15	38	82	10	8
E07.1	High	30	34	36	31	60	9

Table S3. Dominant taxa for the biofilms assessed by PAM fluorometry. The top three most abundant taxa for Cyanobacteria (Cyano), Chlorophyta (Green; i.e. green algae), and Bacillariophyta (Diatom) are indicated based on Palmer-Maloney microscope cell counts across mesocosms. Microscope-based cell counts were made on on aliquots of periphyton slurries processed from destructive sampling of ten pieces of gravel (high light) and two tiles (low light) per mesocosm. (The PAM fluorometry measurements presented in the manuscript were taken two days later on mesocosm biofilms that had not been destructively sampled. Note also, that high light biofilm substrates included both gravel and tile for the PAM fluorometry.)

Lighting	Rank	Cyano	Green	Diatom
Low	1	Phormidium	Stigeoclonium	Cocconeis
Low	2	unknown filamentous	colonial coccoid	Melosira
Low	3	colonial coccoid	unknown filamentous	Fragilaria
High	1	Phormidium	Stigeoclonium	Fragilaria
High	2	Planktolyngbya	Mougeotia	Cocconeis
High	3	Lyngbya	Scenedesmus	Melosira

2. Supplementary Figures

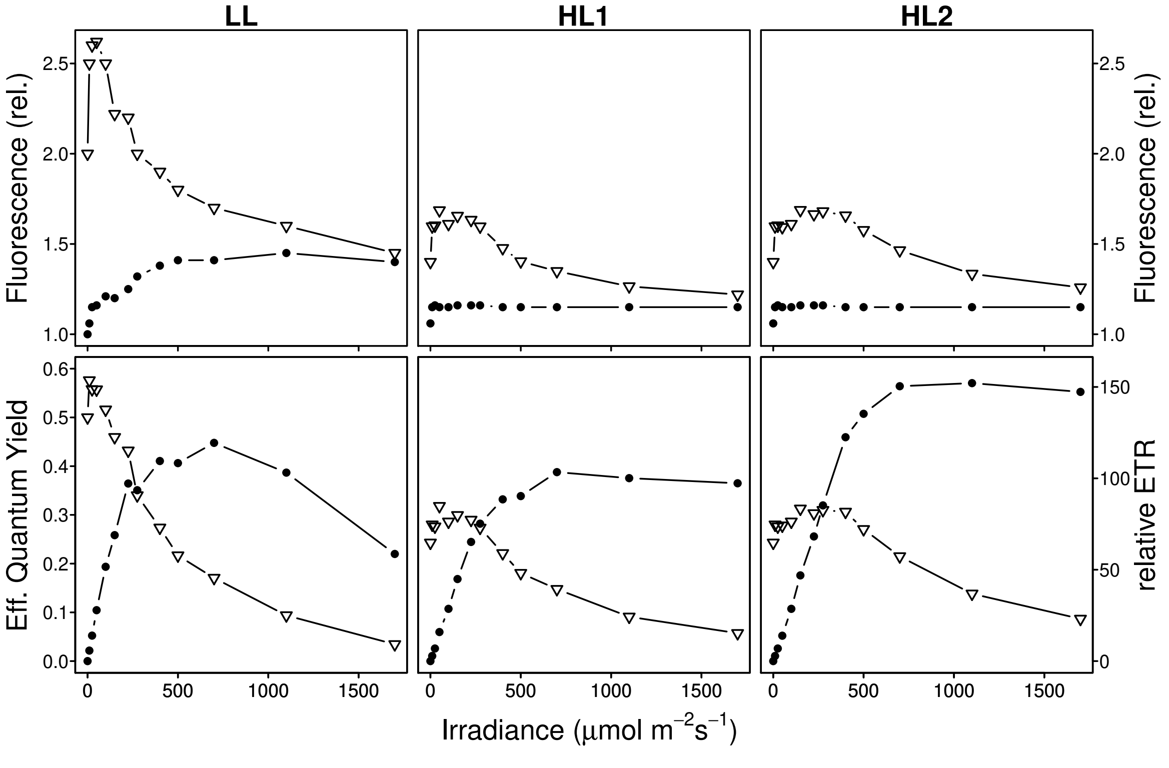

Figure S1. Irradiance response curves for simulated LL- and HL-adapted algae. Top panel shows the maximum (F_m'; open triangles) and minimum (F; filled circles) fluorescence profiles (relative units). Bottom panel shows the effective quantum yield (Φ_{PSII}, dimensionless; open triangles) and relative electron transport rate ($rETR$, µmol m^{-2} s^{-1} electrons; filled circles).

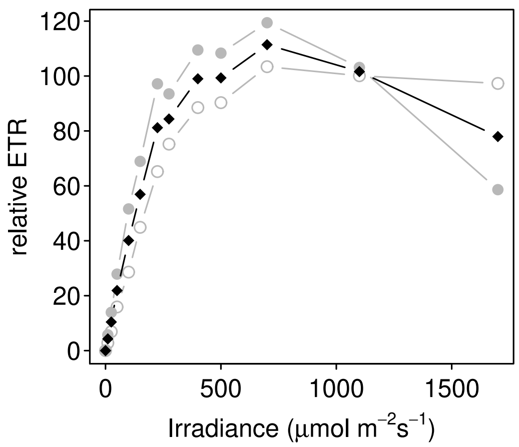

(a) HL1LL

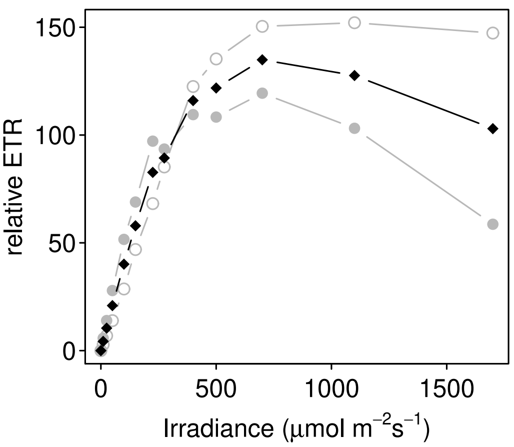

(b) HL2LL

Figure S2. The *rETR* profiles utilized for simulating the two photosynthetically heterogeneous biofilms (HL1LL and HL2LL). To estimate true values for the heterogeneous biofilm comprised of an upper HL-adapted layer and lower LL-adapted layer (in equal proportions), the homogeneous *rETR* profiles were averaged, then the photosynthetic parameters were estimated by the hyperbolic tangent model. In each subfigure, the HL-adapted *rETR* profile is indicated by the unfilled gray circles and the LL-adapted *rETR* profile is indicated by the filled gray circles (these match the *rETR* profiles in Figure S1). The *rETR* profile for the heterogeneous biofilm is indicated by the black diamonds.

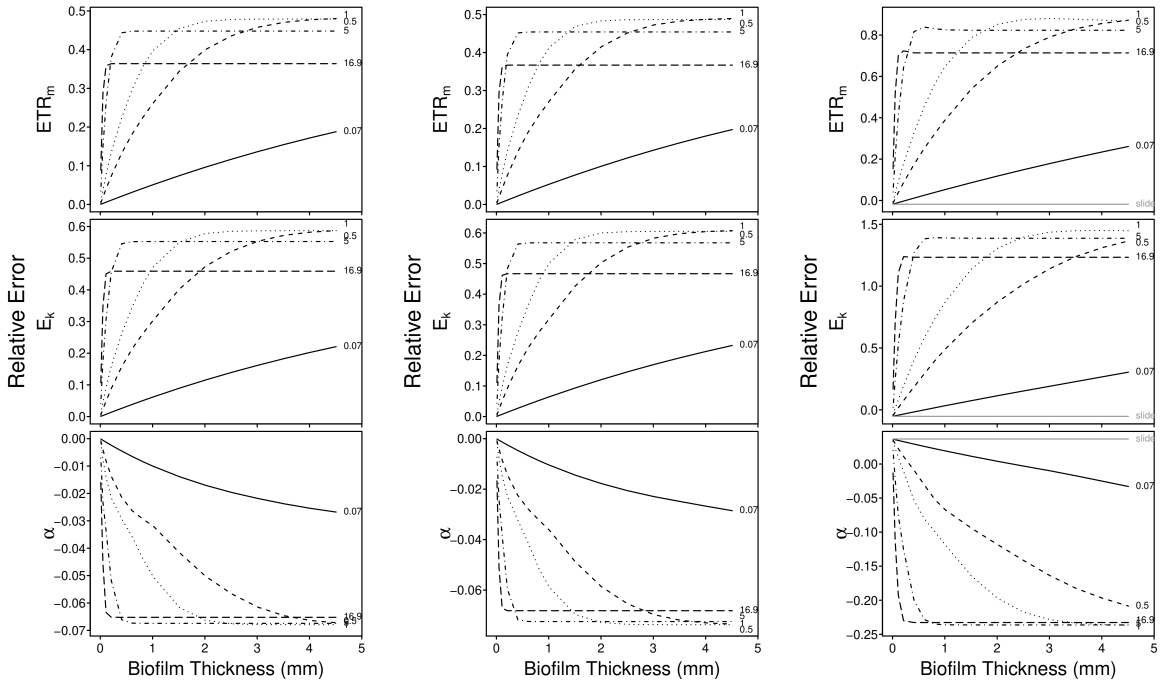

(a) HL1 (b) HL2 (c) HL2LL

Figure S3. Relative error in estimates of ETR_m, E_k and α for (a,b) photophysiologically homogeneous biofilms of varying thickness and light attenuation; and (c) a photophysiologically heterogeneous biofilm of varying thickness and light attenuation. Specifically, the HL1 community is modeled in a, the HL2 community is modeled in b and the HL2LL community is modeled in c (see also Figure 1 for the LL and HL1LL communities). True values for each parameter can be found in Table 1. Each community was simulated over 15 biofilm thicknesses (range 0.01 mm to 4.51 mm) and 5 downwelling attenuation coefficients (k_d: 0.07 mm^{-1}, solid; 0.5 mm^{-1}, dashed; 1 mm^{-1}, dotted; 5 mm^{-1}, dotdash; and 16.9 mm^{-1}, longdash). For the homogeneous communities (e.g., a,b) the slide method is assumed to be completely unbiased. For the heterogeneous communities (e.g., c), however, averaging of the fluorescence yields from the upper (HL) and lower (LL) layers results in slight bias in the slide-based estimates (indicated in c, solid gray).

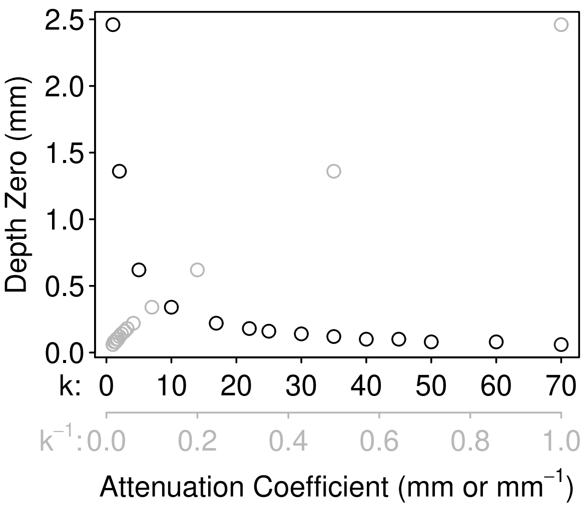

Figure S4. We also considered a critical depth, or depth zero (z_0), below which the surface irradiance decayed to approximately 0. This depth had an approximately linear relationship with the inverse of k_d. Here we plot depth zero (z_0) or the *effective* biofilm thickness against the downwelling attenuation coefficient k_d (black) and its inverse k_d^{-1} (gray). To create this plot, ETR_m was estimated following the depth-integration model for a range of attenuation coefficients and biofilm thicknesses. For each k_d, the estimated ETR_m increased initially with biofilm thickness then plateaued (see e.g., Figures 1 and S3); the thickness at this transition point was considered z_0 and is plotted here. As k_d becomes large, z_0 approaches zero (black circles). For the inverse of k_d, or k_d^{-1}, the relationship with z_0 is approximately linear (gray circles). This pattern is predictable from the model for the exponential decay of irradiance: $E(z, k_d) = E_0 e^{-zk_d}$, where $E(z, k_d)$ is the effective irradiance at depth z given k_d and E_0 is the surface irradiance. Assume some critical value E_{crit} for $E(z, k_d)$ where we consider the surface irradiance to have decayed to essentially 0, such as $0.1E_0$, then we can write $E(z, k_d) = 0.1E_0 = E_0 e^{-z_0 k_d}$ (and denoting the depth where this occurs as z_0), which can be rearranged to $\ln(0.1E_0/E_0) = -z_0 k_d$, and finally $z_0 = -\ln(0.1)k_d^{-1}$.

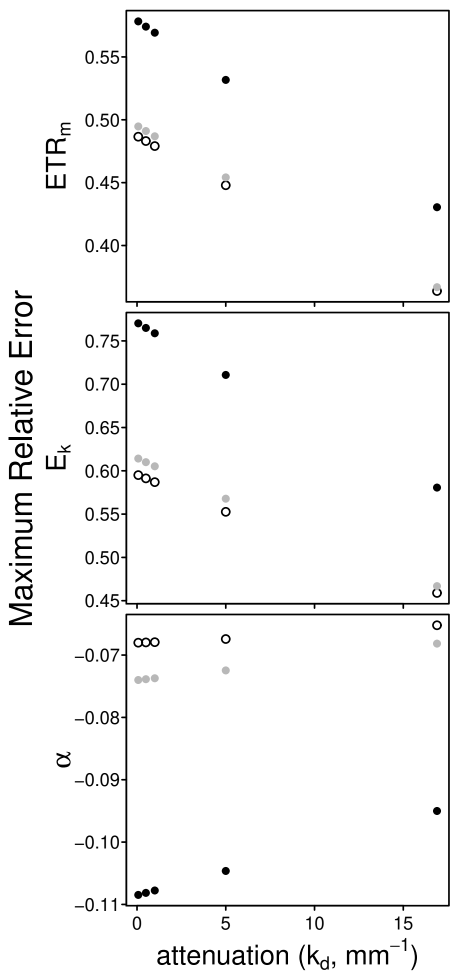

Figure S5. Maximum bias predicted from depth-integration effects was linearly dependent on the downwelling attenuation coefficient (k_d) and the community (i.e. the fluorescence yield profiles over irradiance; Figure S1). Here we plot the maximum bias estimated for each value of k_d; the maximum bias was estimated by simulating the depth integration effects at a thickness greater than the critical depth (z_0; Figure S4) for each value of k_d. Maximum bias was estimated for each homogeneous community (LL, filled; HL1, open; HL2, shaded). The y-intercept for each linear relationship was directly proportional to the initial slope of each Φ_{PSII} profile (i.e. the true α for each homogeneous biofilm; Table 1 and Figure S1).

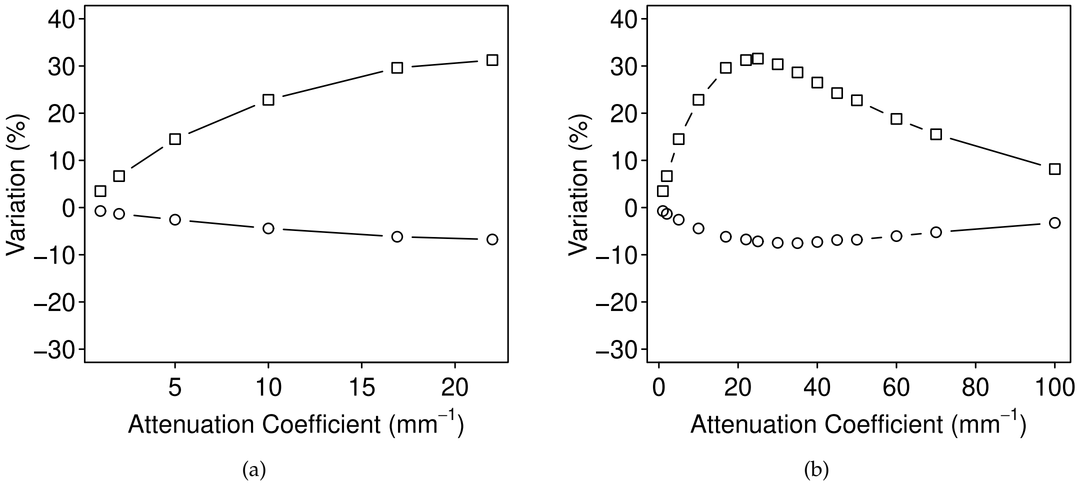

Figure S6. Impact of an increasing attenuation coefficient k on ETR_m and α (plotted as percent variation from true, depth-independent values). These results were based on modeled depth-integration effects for a biofilm of 0.04 mm thickness with the LL-adapted photophysiology. (a) replicates Figure 5b in Serôdio [1] for k_d ranging from 1 mm^{-1} to 22 mm^{-1} and demonstrates the general pattern of increasing over/underestimation as k_d increases [minor differences can be attributed to our use of the hyperbolic tangent model with no photoinhibition [2], whereas Serôdio [1] used the exponential model with photoinhibition [3]]. This pattern will hold until k_d becomes so large that the *effective* biofilm thickness approaches zero and the measured photophysiological parameters will approach the true depth-independent values. This is demonstrated in (b) where we have simply increased the model range of k_d from a maximum of 22 mm^{-1} to a maximum of 100 mm^{-1}.

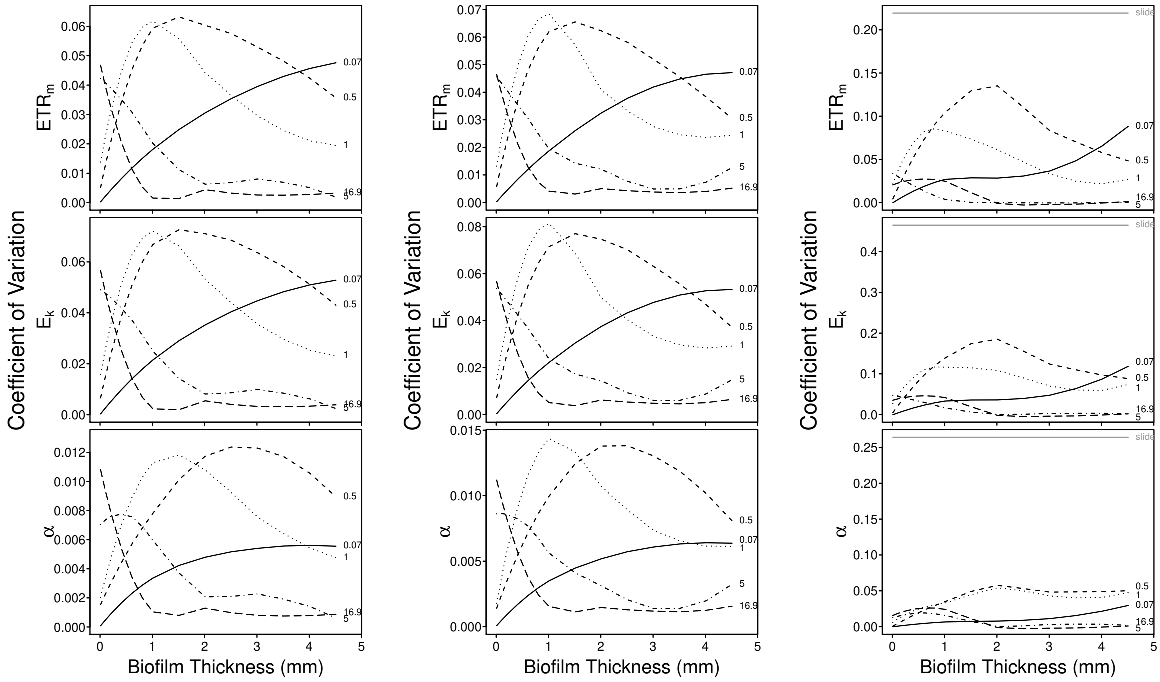

(a) HL1 (b) HL2 (c) HL2LL

Figure S7. Coefficient of variation for estimates of ETR_m, E_k and α in (a,b) photophysiologically homogeneous biofilms of varying thickness and light attenuation; and (c) a photophysiologically heterogeneous biofilm of varying thickness and light attenuation. Specifically, the HL1 community is modeled in a, the HL2 community is modeled in b and the HL2LL community is modeled in c (see also Figure 4 for the LL and HL1LL communities). Each community was simulated over 15 biofilm thicknesses (range 0.01 mm to 4.51 mm) and 5 downwelling attenuation coefficients (k_d: 0.07 mm^{-1}, solid; 0.5 mm^{-1}, dashed; 1 mm^{-1}, dotted; 5 mm^{-1}, dotdash; and 16.9 mm^{-1}, longdash). To assess depth-integration effects on precision (intact-biofilm method; black lines), at each modeled thickness (per community and k_d) a random sample of $n = 500$ was generated based on the given thickness and a CV of 40%. Precision was estimated using the standard formula for CV. To assess potential subsampling effects on precision (slide method; gray lines), the CV was estimated based on three subsamples: (1) 50% of the HL and 50% of the LL layer; (2) 100% of the HL layer; and (3) 100% of the LL layer. Accordingly, for the homogeneous biofilms the CV for the slide method was assumed 0 (not plotted in a). (Note, the lines plotted were smoothed by LOESS using the *loess* function in R.)

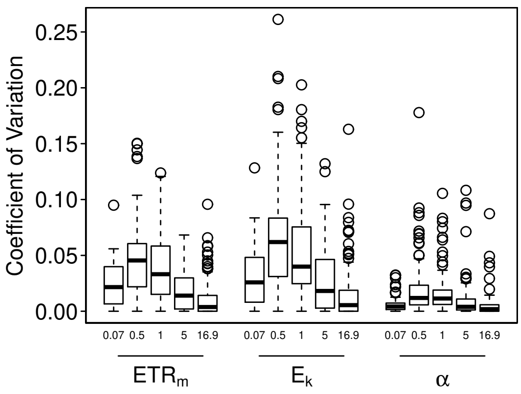

(a) all

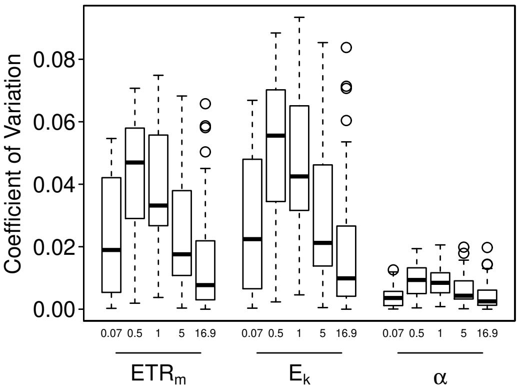

(b) homogeneous

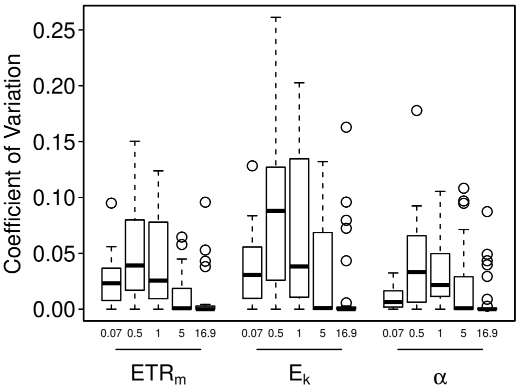

(c) heterogeneous

Figure S8. Coefficient of variation boxplots for estimates of ETR_m, E_k and α relative to the downwelling attenuation coefficient across (a) all simulated biofilms; (b) photophysiologically homogeneous biofilms; and (c)photophysiologically heterogeneous biofilms. Mean precision tended to be higher for very small k_d (e.g., $k_d = 0.07$ mm^{-1}) and for very large k_d (e.g., $k_d > 5$ mm^{-1}). For very small k_d, the rate-of-change in parameter bias was slower with thickness (e.g., compare initial regions of the curves in Figures 1 and S3), resulting in less variability in parameter estimates because of depth-integration effects over a spatially-variable biofilm (i.e. with variable thickness). For very large k_d, the rapid attenuation of light resulted in only the uppermost layers of the biofilm being activated and contributing to the depth-integration effects despite a spatially-variable biofilm. The box-and-whiskers indicate the medians (central bar), first and third quartiles (box boundaries), and lower and upper extremes ("whiskers") for each group; outliers are plotted as open circles.

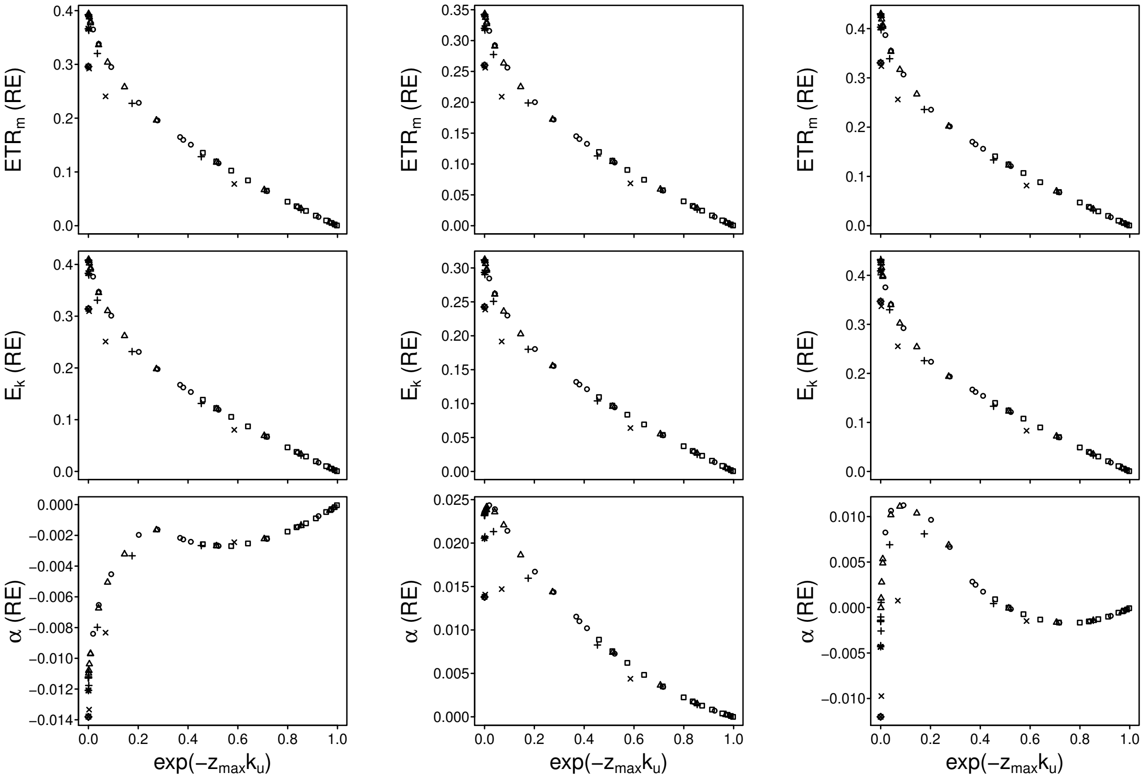

(a) (b) (c)

Figure S9. Comparison of predicted depth-integration effects for three biofilm profiles. The original profiles were obtained using the slide technique and thus approximate thin film, depth-independent measurements. For each profile we simulated depth-integration effects for five downwelling attenuation coefficients and fifteen biofilm thicknesses. We plot the relative error (RE) for each parameter against the exponential of the biofilm thickness (z_{max}) and the upwelling attenuation coefficient (k_u). For all three biofilm simulations, ETR_m and E_k were always overestimated because of the depth-integration effects. In (a), α was always underestimated—similar to our initial simulations and those of Serôdio [1]. In (b), α was almost always overestimated (specifically 99% overestimated and 1% underestimated)—this aligns with the majority of our empirical comparisons of paired slide-based and intact-biofilm-based estimates. In (c), α was overestimated for some $z_{max}k_u$ combinations (29%) and underestimated for other $z_{max}k_u$ combinations (71%). (Plot symbols indicate the downwelling attenuation coefficient (k_d): 0.07 mm^{-1}, squares; 0.5 mm^{-1}, circles; 1 mm^{-1}, triangles; 5 mm^{-1}, crosses; and 16.9 mm^{-1}, diamonds. The upwelling coefficient k_u was scaled relative to k_d following Serôdio [1]: $k_u = k_d(53.5/16.9)$. The relationship was stronger for k_u, as opposed to k_d, hence k_u is plotted here.)

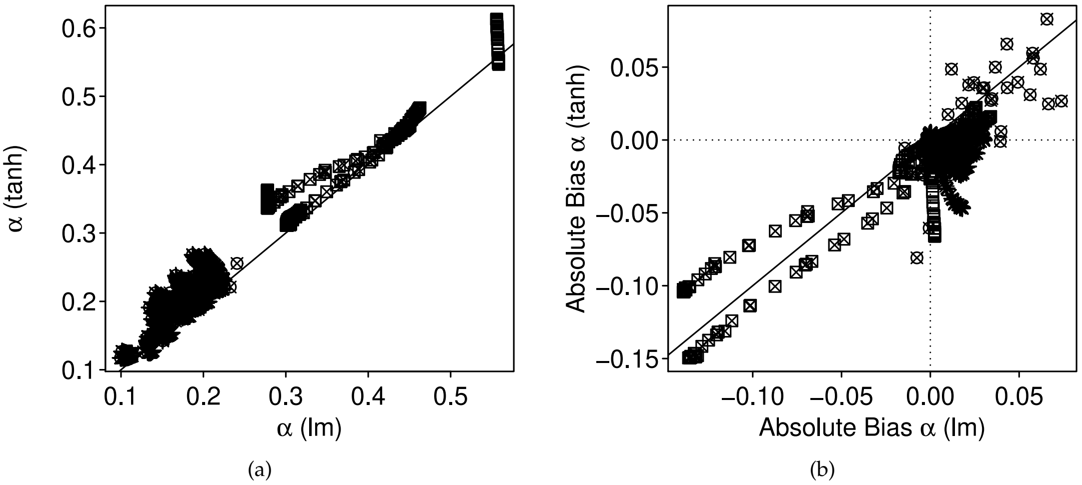

Figure S10. Comparison of estimates of α between the hyperbolic tangent model (tanh) and simple linear regression (lm). For the hyperbolic tangent model, α was estimated using the full dataset. For the simple linear regression α was estimated using only the first four observations (maximum irradiance typically less than 95 μmol m^{-2} s^{-1}). In (a), the actual estimates are compared—estimates from the two methods are strongly correlated ($r = 0.97$, $p < 0.001$). The estimate from the hyperbolic tangent model, however, is almost always higher. In (b), the absolute bias (depth-dependent value − depth-independent value) introduced by depth-integration is compared between the two methods of estimation. Both estimates are biased when there are depth-integration effects and can result in over or underestimation of α. [Solid lines indicate 1:1 relationships; dotted lines (b) indicate zero bias. Plotting symbols are as follows: open circle = slide (in b only and overlain by depth-integrated estimates), circle with cross = intact biofilm, open square = depth-integrated homogeneous biofilms, square with cross = depth-integrated heterogeneous biofilms, asterisk = depth-integrated slide.]

References

1. Serôdio, J. Analysis of variable chlorophyll fluorescence in microphytobenthos assemblages: implications of the use of depth-integrated measurements. *Aquatic Microbial Ecology* **2004**, *36*, 137–152. doi:10.3354/ame036137.
2. Jassby, A.D.; Platt, T. Mathematical formulation of the relationship between photosynthesis and light for phytoplankton. *Limnology and Oceanography* **1976**, *21*, 540–547. doi:10.4319/lo.1976.21.4.0540.
3. Platt, T.; Platt, T, C.L.; Harrison, W.G. Photoinhibition of photosynthesis in natural assemblages of marine phytoplankton. *Journal of Marine Research* **1980**, *38*, 687–701.
